# Cross-Activation of Two Nitrogenase Gene Clusters by CnfR1 or CnfR2 in the Cyanobacterium Anabaena variabilis

**DOI:** 10.1128/Spectrum.01060-21

**Published:** 2021-10-06

**Authors:** Brenda S. Pratte, Teresa Thiel

**Affiliations:** a Department of Biology, University of Missouri-St. Louis, St. Louis, Missouri, USA; College of New Jersey

**Keywords:** nitrogenase, nitrogen fixation, nitrogen regulation, cyanobacteria, heterocyst, CnfR

## Abstract

In Anabaena variabilis, the *nif1* genes, which are activated by CnfR1, produce a Mo-nitrogenase that is expressed only in heterocysts. Similarly, the *nif2* genes, which are activated by CnfR2, make a Mo-nitrogenase that is expressed only in anaerobic vegetative cells. However, CnfR1, when it was expressed in anaerobic vegetative cells under the control of the *cnfR2* promoter or from the Co^2+^-inducible *coaT* promoter, activated the expression of both *nifB1* and *nifB2*. Activation of *nifB2*, but not *nifB1*, by CnfR1 required NtcA. Thus, expression of the *nif1* system requires no heterocyst-specific factor other than CnfR1. In contrast, CnfR2, when it was expressed in heterocysts under the control of the *cnfR1* promoter or from the *coaT* promoter, did not activate the expression of *nifB1* or *nifB2*. Thus, activation of the *nif2* system in anaerobic vegetative cells by CnfR2 requires additional factors absent in heterocysts. CnfR2 made from the *coaT* promoter activated *nifB2* expression in anaerobic vegetative cells grown with fixed nitrogen; however, oxygen inhibited CnfR2 activation of *nifB2* expression. In contrast, activation of *nifB1* and *nifB2* by CnfR1 was unaffected by oxygen. CnfR1, which does not activate the *nifB2* promoter in heterocysts, activated the expression of the entire *nif2* gene cluster from a *nifB2*::*nifB1*::*nifB2* hybrid promoter in heterocysts, producing functional Nif2 nitrogenase in heterocysts. However, activity was poor compared to the normal Nif1 nitrogenase. Expression of the *nif2* cluster in anaerobic vegetative cells of *Nostoc* sp. PCC 7120, a strain lacking the *nif2* nitrogenase, resulted in expression of the *nif2* genes but weak nitrogenase activity.

**IMPORTANCE** Cyanobacterial nitrogen fixation is important in the global nitrogen cycle, in oceanic productivity, and in many plant and fungal symbioses. While the proteins that mediate nitrogen fixation have been well characterized, the regulation of this complex and expensive process is poorly understood in cyanobacteria. Using a genetic approach, we have characterized unique and overlapping functions for two homologous transcriptional activators CnfR1 and CnfR2 that activate two distinct nitrogenases in a single organism. We found that CnfR1 is promiscuous in its ability to activate both nitrogenase systems, whereas CnfR2 depends on additional cellular factors; thus, it activates only one nitrogenase system.

## INTRODUCTION

Biological nitrogen fixation is the reduction of atmospheric dinitrogen to ammonium, mediated by the oxygen-sensitive enzyme nitrogenase. Nitrogen fixation is found only in some bacterial and archaeal strains, but it is essential for the productivity of the oceans and is important for the growth of many plants that benefit from nitrogen fixation by rhizosphere bacteria or by symbiotic associations with nitrogen-fixing bacteria (1, 2). Cyanobacteria play a major role in nitrogen productivity in the oceans and form symbiotic associations with many plants and fungi (3, 4).

All cyanobacteria produce oxygen from photosynthesis; therefore, nitrogen-fixing strains must protect nitrogenase from oxygen. Nitrogen fixation is separated from photosynthesis either spatially in specialized microoxic cells or temporally when cells are not producing oxygen by photosynthesis (5–8). Cyanobacteria in the *Nostocaceae* differentiate specialized nitrogen-fixing cells called heterocysts but only under conditions of nitrogen starvation. Heterocysts provide a microoxic environment to protect nitrogenase, allowing the cyanobacterial filament to fix nitrogen in air (9–12). The microoxic environment of the heterocysts results from their cell wall structure, their lack of oxygen-evolving photosystem II, and their high rate of respiration (13–17). All heterocyst-forming cyanobacteria express a Mo-nitrogenase in heterocysts, but some strains also have a heterocyst-specific V-nitrogenase that is made only under conditions of Mo deficiency (18, 19).

The genes for nitrogen fixation in cyanobacteria are well conserved and typically include a few clusters of *nif* genes, some of them comprising over a dozen genes (20). These large clusters of genes have been best characterized in Anabaena variabilis ATCC 29413 (19), Leptolyngbya boryana (21, 22), and *Cyanothece* sp. (7, 23). *A. variabilis* is unusual because it has two *nif*-encoded Mo-nitrogenases. The *nif1* genes are expressed only in heterocysts, while the *nif2* genes are expressed only in anaerobic vegetative cells (19, 20, 24). The expression of the *nif1* genes depends on the differentiation of heterocysts, a complex developmental process that requires a carefully regulated cascade of regulatory factors, including NtcA, HetR, NrrA, DevH, and other proteins (9, 11, 12, 25). NtcA, which senses changes in the cellular carbon-to-nitrogen ratio, is important not only in heterocyst differentiation but also in the global cellular nitrogen response (26, 27). While the *nif1* and *nif2* gene clusters are expressed in different cell types and under different conditions, they have a very similar gene organization comprising a large cluster of contiguous genes, *nifB*, *nifS*, *nifU*, *nifH*, *nifD*, *nifK*, *nifE*, *nifN*, *nifX*, *nifW*, *hesA*, and *hesB*, and a few genes of unknown function. The *nif1* and *nif2* gene clusters are primarily under the control of the promoter upstream of *nifB*, although *nifU1* and *nifE1* have weak promoters within the genes and *hesA1* has its own strong promoter (20, 28, 29). A major difference between the two clusters is the interruption of the *nifD1* gene by an 11-kb element that must be excised during heterocyst differentiation (30).

The large cyanobacterial *nif* clusters are regulated by the transcriptional activator CnfR. CnfR proteins all share a C-terminal XRE-type DNA-binding domain and two N-terminal 4Fe-4S-binding sites, similar to bacterial ferredoxins (21, 31–33). The *cnfR* gene was first described in *Nostoc* sp. PCC 7120 as *patB*, a gene that affected heterocyst pattern formation (32). In *Nostoc* sp. PCC 7120, the *patB* (*cnfR*) gene is expressed exclusively in heterocysts, and a deletion mutant grew very poorly in the absence of fixed nitrogen (31). The *cnfR1* gene of *A. variabilis* is very similar to *cnfR* (*patB*) in *Nostoc* sp. PCC 7120. In the nonheterocystous strain L. boryana, deletion of either the conserved CnfR N-terminal iron-sulfur cluster-binding region or the C-terminal DNA-binding domain abolished expression of *nifB* (21, 22).

In *A. variabilis*, CnfR1 activates expression of the *nifB1* promoter only in heterocysts, and CnfR2 activates expression of the *nifB2* promoter only in anaerobic vegetative cells (33). Deletion of *cnfR1* or *cnfR2* prevents the expression of *nifB1* or *nifB2*, respectively. Loss of *cnfR1* has little effect on the expression of *hesA1*, which has its own promoter, whereas *hesA2* expression is driven by the *nifB2* promoter and is therefore inhibited by loss of *cnfR2* (29, 33). Expression of *cnfR2* in anaerobic vegetative cells requires NtcA; hence, an *ntcA* mutant does not make the Nif2 nitrogenase in anaerobic vegetative cells (20, 33, 34).

A region 300 to 600 bp upstream of the transcription start sites of *nifB1* and *nifB2* and upstream of the promoter of *nifB* in L. boryana have three conserved *cis*-acting elements that are thought to be the binding sites for CnfR proteins (22, 35). In *A. variabilis*, the loss of either the first or the second conserved site decreases transcription of *nifB1* by about 50%, and loss of both of these conserved sites leads to a 75% reduction in *niB1* expression (35). The transcription start sites for both the *nifB1* and *nifB2* promoters have a canonical “extended −10 promoter” (35, 36) but no −35 region, indicating that they are a type 2 promoter, which often requires an alternative sigma factor (37).

Several cyanobacterial sigma factors respond to various types of stress (38–40). In *Nostoc* sp. PCC 7120, SigB and SigC play a role in the expression of some NtcA-dependent, nitrogen-responsive genes (37), and *sigC*, *sigE*, and *sigG* expression are increased in heterocysts (41, 42). Both SigC (42) and SigE (43) are involved in the expression of *nif* genes in heterocysts, but they are not essential. HetR, the central activator of heterocyst differentiation, increases the expression of SigC. Overall, the data indicate that *sigB*, *sigC*, *sigD*, and *sigE* likely have roles, which may overlap, in the expression of nitrogen-regulated genes and possibly in nitrogen fixation.

We are interested in the mechanism of CnfR-mediated activation of expression of *nifB* and the possible role of other factors in *nifB* transcription. Since *A. variabilis* has two CnfR proteins that act in different cell types to activate two *nifB* promoters, we determined whether these two CnfR proteins could activate the noncognate *nifB* promoter, how cell type affected *nifB* activation, and how environmental conditions or factors, such as NtcA, NrrA, or HetR, affected CnfR-mediated activation of *nifB1* and *nifB2*. We determined how hybrid CnfR1-CnfR2 proteins and a hybrid *nifB2*-*nifB1* promoter affected *nifB* transcription. In addition, we showed that the *nif2* genes of *A. variabilis* were expressed in *Nostoc* sp. PCC 7120, producing the Nif2 nitrogenase in anerobic vegetative cells.

## RESULTS

### CnfR proteins.

CnfR proteins are present in all sequenced nitrogen-fixing cyanobacteria, and strains with two Mo-nitrogenase gene clusters have two copies of *cnfR* (Fig. S1 and Table S1 in the supplemental material). Cyanobacterial CnfR proteins cluster into four phylogenetic groups, including one group of CnfR1 proteins in heterocyst-forming cyanobacteria and another that includes the CnfR2 proteins. CnfR2 proteins are more closely related to the CnfR protein in *Chroococcidiopsis thermalis* PCC 7203 than to CnfR proteins in heterocystous cyanobacteria lacking a *nif2* system (Fig. S1) (33). The sequence similarities between CnfR proteins in the most divergent strains are about 60%. In *A. variabilis*, the CnfR1 and CnfR2 proteins share 61% identity and 80% overall similarity, but they are more similar to each other than to the CnfR proteins of most nonheterocystous cyanobacteria (Fig. S1). All CnfR proteins have an N-terminal Fe-S-binding domain and a C-terminal helix-turn-helix (HTH) domain. The C-terminal HTH domains of CnfR1 and CnfR2 in *A. variabilis* share 87% similarity, while the N-terminal Fe-S-binding domains share only 73% similarity (33).

### The role of the DNA-binding domains in activation of *nifB1* and *nifB2* in heterocysts.

In *A. variabilis*, CnfR1 activates *nifB1*, the first gene in the large *nif* cluster, in heterocysts, while CnfR2 activates *nifB2* in anaerobic vegetative cells starved for fixed nitrogen. Therefore, the promoters that drive expression of *cnfR1* versus *cnfR2* are only activated in heterocysts or anaerobic vegetative cells, respectively (33). We were interested in the ability of CnfR1, CnfR2, and hybrid CnfR1-CnfR2 proteins to cross regulate expression of *nifB1* and *nifB2*; therefore, we expressed them from promoters that drove their expression under conditions in which *cnfR1* and *cnfR2* are not normally produced.

**FIG 1:**
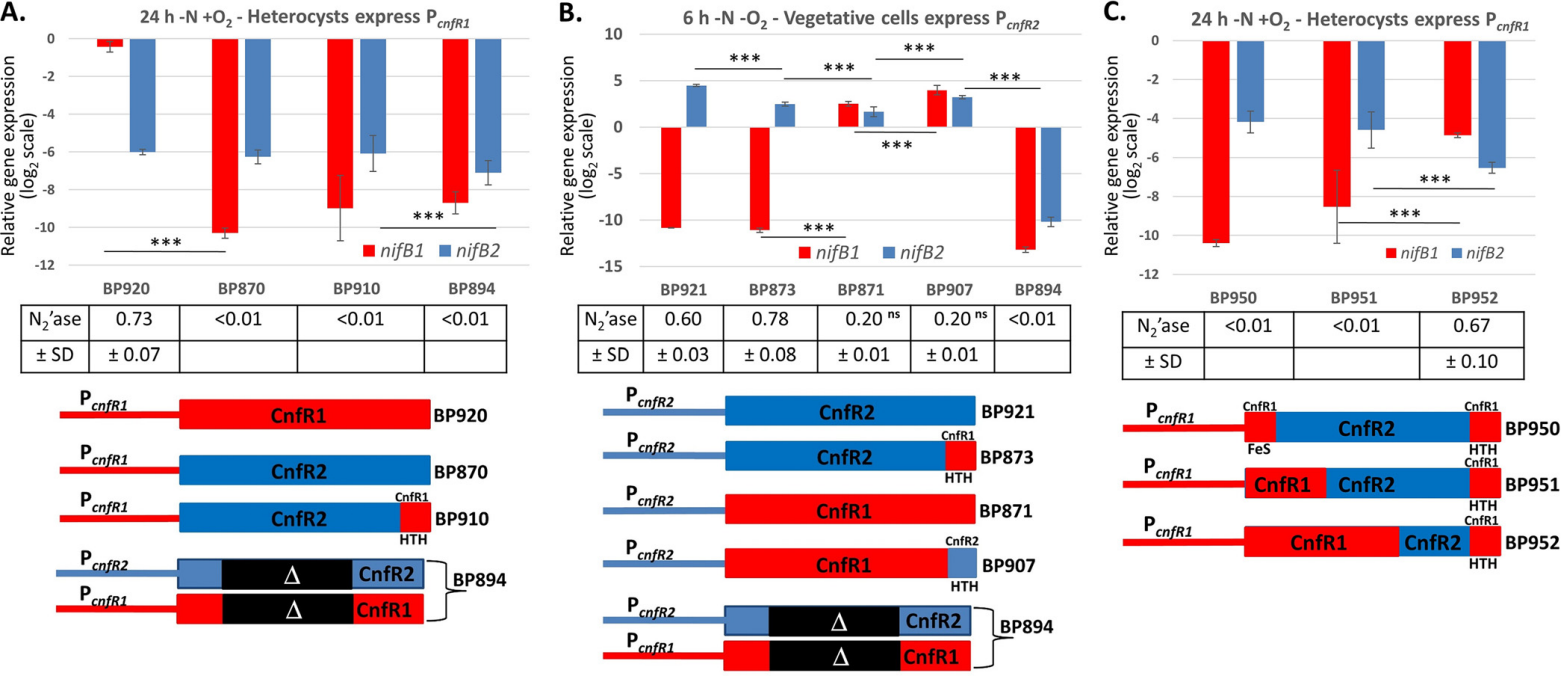
Cell-type-specific expression of *nifB1* and *nifB2* by CnfR1 and CnfR2, respectively. Expression of *nifB1* or *nifB2* by CnfR1, CnfR2, or a hybrid CnfR protein, driven by either the *cnfR1* or *cnfR2* promoter as indicated, was determined by RT-qPCR. (A, C) Cells were grown 24 h under −N +O_2_ conditions, leading to formation of heterocysts that activate the *cnfR1* promoter. (B) Cells were grown 6 h under −N −O_2_ conditions, leading to vegetative cells that activate the *cnfR2* promoter. Expression of *nifB1* and *nifB2* was normalized to *rnpB*, and the values on the *y* axis represent the relative log_2_ fold differences in expression. Nitrogenase activity (N_2_'ase) is expressed as nmoles of ethylene OD_720_^−1 ^min^−1^. Statistical analysis: graphs, ***, *P < *0.001; nitrogenase, all differences in values were significant (*P < *0.001) except the two labeled nonsignificant (ns; *P ≥* 0.05). The horizontal bars below the *P* values provide statistical comparisons for the means of the two values immediately below the ends of the bar and do not include values between these two.

CnfR1 made under the control of its own promoter in heterocysts (strain BP920) at 24 h after nitrogen step-down activated the expression of *nifB1* more than 200-fold compared to the Δ*cnfR1*/Δ*cnfR2* control strain (BP894) but did not activate the expression of *nifB2* (Fig. 1A). CnfR2 made under the control of the *cnfR1* promoter in heterocysts (BP870) at 24 h after nitrogen step-down was induced 5-fold compared to anaerobic vegetative cells (Fig. S2) but failed to activate expression of either *nifB1* or *nifB2* (Fig. 1A). Replacement of the C-terminal HTH DNA-binding domain of CnfR2 by the HTH domain of CnfR1 (CnfR2-HTH_CnfR1_) in BP910 did not allow CnfR2 to activate *nifB1* or *nifB2* in heterocysts (Fig. 1A). This suggests that the difference in the DNA-binding domains of CnfR1 versus CnfR2 was not responsible for the inability of CnfR2 to activate *nifB1* or *nifB2* in heterocysts.

### The role of the DNA-binding domains in activation of *nifB1* and *nifB2* in vegetative cells.

CnfR2 made under the control of its own promoter (BP921) in anaerobic vegetative cells 6 h after nitrogen step-down activated the expression of *nifB2* several thousand-fold (quantification cycle [ΔΔ*^Cq^*] = 14) compared to the Δ*cnfR1*/Δ*cnfR2* control strain (BP894). In contrast, CnfR2 did not activate the expression of *nifB1* (Fig. 1B). Replacement of the HTH domain of CnfR2 by the HTH domain of CnfR1 (CnfR2-HTH_CnfR1_) in BP873 decreased the expression of *nifB2* about 4-fold but did not increase expression of *nifB1* (Fig. 1B). Expression of CnfR1, driven by the *cnfR2* promoter (BP871), in anaerobic vegetative cells 6 h after nitrogen stepdown was induced 3-fold compared to cells grown aerobically for 24 h (Fig. S2), but it strongly activated the expression not only of *nifB1* but also of *nifB2* (Fig. 1B). Expression of CnfR1-HTH_CnfR2_ driven by the *cnfR2* promoter (BP907) increased both *nifB1* and *nifB2* expression about 3-fold compared to CnfR1 driven by the *cnfR2* promoter (Fig. 1B). Thus, CnfR2 can activate only *nifB2* in anaerobic vegetative cells, while CnfR1 can activate *nifB1* and *nifB2*. Replacement of the DNA-binding domain of CnfR1 with the similar region of CnfR2 improved activation of both the *nifB1* and *nifB2* promoters by CnfR1 in anaerobic vegetative cells, where it is not normally expressed.

### Expression of nitrogenase in vegetative cells.

Although *nifB1* was expressed in anaerobic vegetative cells of BP871 and BP907 at 6 h after nitrogen step-down (Fig. 1B), the 11-kb excision element interrupting the *nifD1* gene would not have been excised; hence, no Nif1 nitrogenase would be made. However, activation of *nifB2* by CnfR1 (BP871) or CnfR1-HTH_CnfR2_ (BP907) resulted in Nif2 nitrogenase activity, although activity was lower than in cells that activated *nifB2* using CnfR2 (BP921) or CnfR2-HTH_CnfR1_ (BP873) (Fig. 1B). This decrease could be due to missing proteins whose genes are likely not activated by CnfR1 or due to the production of noncognate Nif1 proteins upstream of the 11-kb excision element in *nifD1*, that is, NifB1, NifS1, NifU1, NifH1, and possibly a truncated NifD1 in addition to the normal NifB2, NifS2, NifU2, NifH2, and NifD2, which may decrease the activity of the Nif2 nitrogenase.

### Effect of hybrid CnfR1/CnfR2 proteins on *nifB1* and *nifB2* expression in heterocysts.

Since CnfR1 activates *nifB1* in heterocysts, but CnfR2 does not, we determined the ability of CnfR1-CnfR2 hybrid proteins to activate *nifB1* in heterocysts. Hybrid proteins that contained mostly CnfR2 sequences (BP870, BP910, BP950, and BP951) showed no activation of *nifB1* in heterocysts and no significant nitrogenase activity (Fig. 1C). However, BP952, with a larger region from CnfR1, increased *nifB1* expression at least 10-fold compared to BP951, which had a smaller region from CnfR1 (Fig. 1C). BP952 also produced high levels of nitrogenase activity, suggesting that the larger CnfR1 region included in BP952 compared to BP951 plays a role in the specific activation of *nifB1*. While CnfR2 (BP870) poorly induced the expression of *nifB2* in heterocysts, two hybrids that included the N-terminal Fe-S domain of CnfR1 (BP950 and BP951) improved *nifB2* expression in heterocysts about 4-fold. However, the hybrid CnfR1-CnfR2 protein that most increased the expression of *nifB1* (BP952) decreased the expression of *nifB2*. It appears that the N-terminal half of CnfR1 is important in determining the specificity of activation of *nifB1* versus *nifB2*.

### Expression of *cnfR1* and *cnfR2* under the control of the *coaT* promoter.

**FIG 2:**
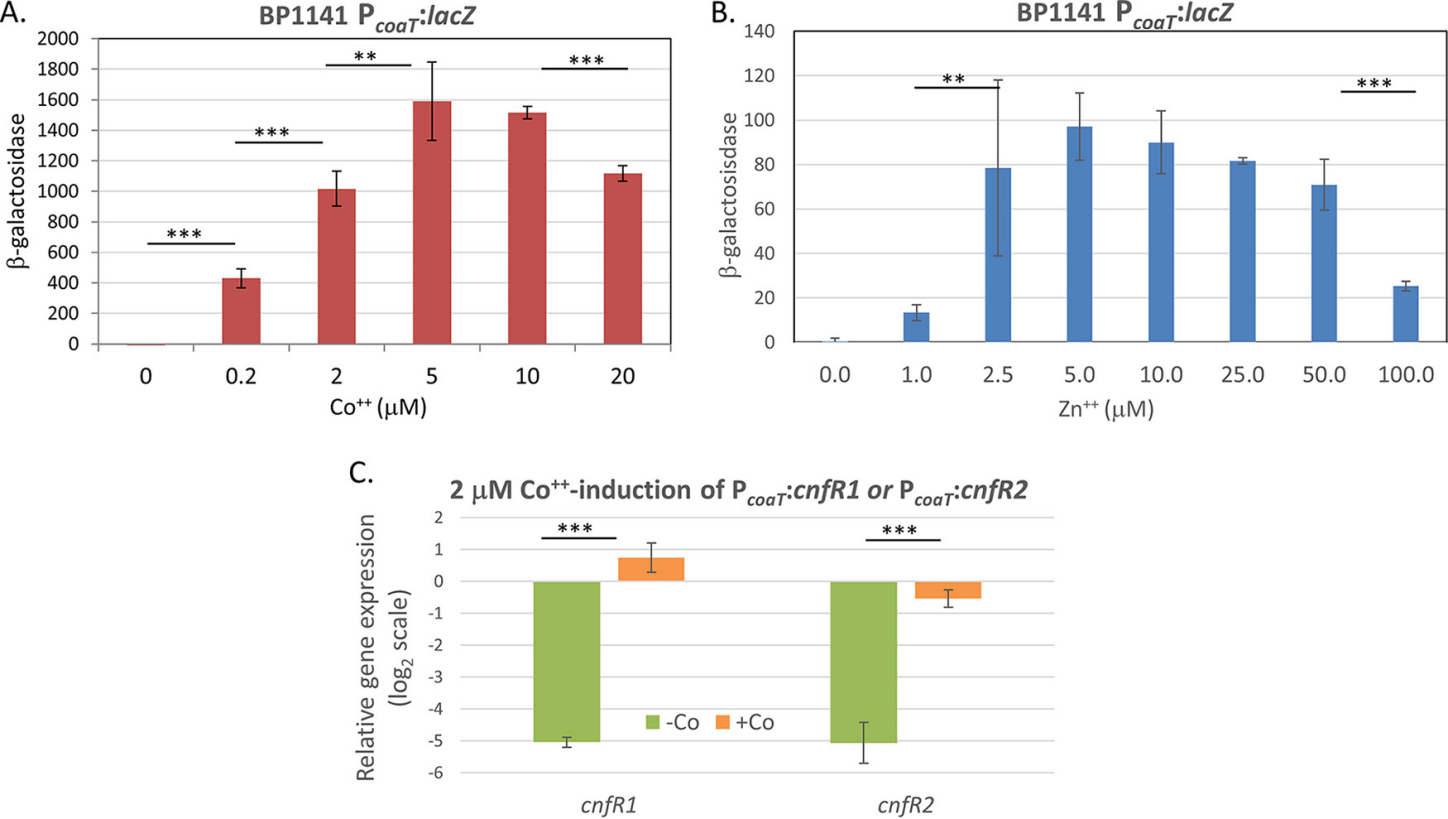
Induction of the P*_coaT_* promoter by Co^2+^ or Zn^2+^. (A) Expression of β-galactosidase in strain BP1141 containing P*_coaT_*::*lacZ* after induction with Co^2+^ for 2 h. (B) The same as A but induced with Zn^2+^. (C) Expression of *cnfR1* or *cnfR2* was determined by RT-qPCR. P*_coaT_*::*cnfR1* (BP1142) was grown for 24 h under −N +O_2_ conditions and induced with 2 μM Co^2+^ for 4 h, and P*_coaT_*::*cnfR2* (BP1143) was grown for 4 h under −N −O_2_ conditions and simultaneously induced with 2 μM Co^2+^. Expression of *cnfR1* and *cnfR2* was normalized to *rnpB*, and the values on the *y* axis represent the relative log_2_ fold differences in expression. *P* values of ≥0.05 are not shown; **, *P < *0.01 or ***, *P < *0.001. The horizontal bars below the *P* values provide statistical comparisons for the means of the two values immediately below the ends of the bar and do not include values between these two. The units for β-galactosidase activity are described in Materials and Methods.

The previous experiments that substituted the promoters of *cnfR1* and *cnfR2* for each other provided information on the activation of *nifB1* and *nifB2* expression only in cell types in which these *cnfR1* and *cnfR2* promoters were expressed. However, we could not determine the effects of fixed nitrogen on the activation of *nifB1* or *nifB2*, since neither the *cnfR1* nor the *cnfR2* promoter is expressed in cells grown with fixed nitrogen; therefore, we tested the *coaT* promoter, which can be induced by Co^2+^ or Zn^2+^ (44–46). We constructed a strain of *A. variabilis* with P*_coaT_*::*lacZ* (BP1141) and measured β-galactosidase activity 2 h after the addition of various concentrations of Co^2+^ or Zn^2+^ (Fig. 2A and B). Concentrations of either metal in the range of 2 to 10 μM induced *lacZ*; however, Co^2+^ induced about 10-fold more β-galactosidase than did Zn^2+^. We induced strains with P*_coaT_*::*cnfR1* (BP1142) or with P*_coaT_*::*cnfR2* (BP1143) with 2 μM Co^2+^, which resulted in a 20- to 50-fold increase in the expression of *cnfR1* or *cnfR2* (Fig. 2C).

**FIG 3:**
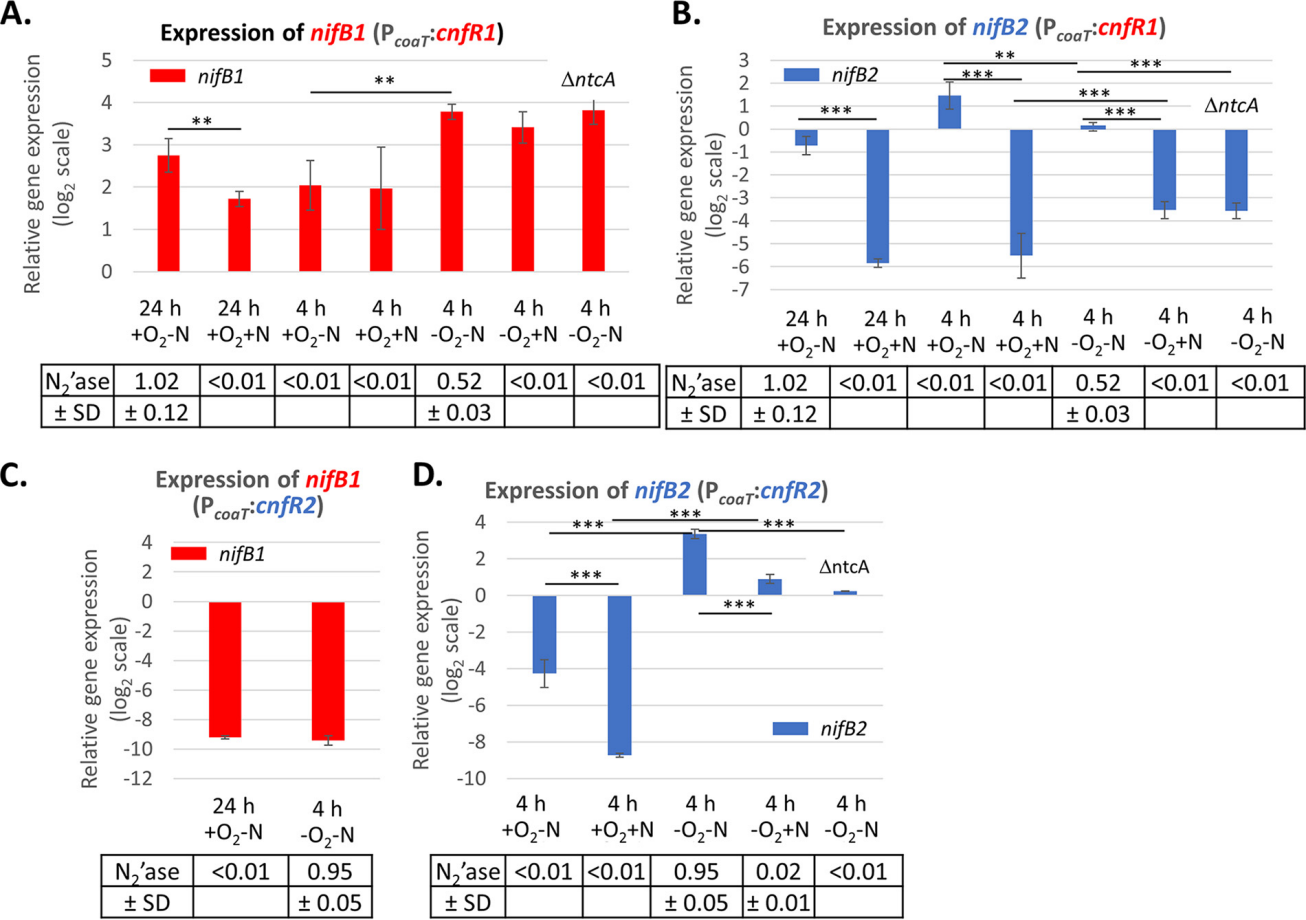
Expression of *nifB1* and *nifB2* by CnfR1 or CnfR2 under various environmental conditions. Expression of *nifB1* (A) or *nifB2* (B) in P*_coaT_*::*cnfR1* (BP1142) and *nifB1* (C) or *nifB2* (D) in P*_coaT_*::*cnfR2* (BP1143) were determined by RT-qPCR in cells grown for 4 h under ±N ±O_2_ conditions or for 24 h under −N +O_2_ conditions (to induce heterocysts). The P*_coaT_* promoter was induced by the addition of Co^2+^ for 4 h before acetylene reduction assays, and cells were harvested for RNA. The P*_coaT_*::*cnfR1* Δ*ntcA* strain was BP7142, and the P*_coaT_*::*cnfR2* Δ*ntcA* strain was BP7143. Expression of *nifB1* and *nifB2* was normalized to *rnpB*, and the values on the *y* axis represent the relative log_2_ fold differences in expression. Nitrogenase activity (N_2_'ase) is expressed as nmoles of ethylene OD_720_^−1 ^min^−1^. Note that nitrogenase data for panels A and B and for C and D are identical because they are from the same experiments. *P* values of ≥0.05 are not shown; **, *P < *0.01 or ***, *P < *0.001. The horizontal bars below the *P* values provide statistical comparisons for the means of the two values immediately below the ends of the bar and do not include values between these two.

Induction of *cnfR1* in strain BP1142 by the addition of Co^2+^ for 4 h starting 20 h after the removal of fixed nitrogen (when heterocysts had already formed) induced about 8-fold more *nifB1* (Fig. 3A) than what was observed in a strain in which *cnfR1* was expressed from its native promoter (compare BP1142 in Fig. 3A with BP920 in Fig. 1A). CnfR1 activated *nifB1* in cells grown with fixed nitrogen (Fig. 3A), indicating that *nifB1* was expressed in vegetative cells. However, *nifB1* expression in filaments with heterocysts (24 h, +O_2_ −N) was about 2-fold higher than in filaments without heterocysts (24 h, +O_2_ +N) (Fig. 3A). Since heterocysts comprise only about 10% of the cells, a 2-fold increase that results from activation only in heterocysts is about a 20-fold increase compared to vegetative cells. Expression of CnfR1 4 h after Co^2+^ induction in vegetative cells grown with or without fixed nitrogen and with or without oxygen resulted in activation of *nifB1* expression (Fig. 3A). While the presence of fixed nitrogen had little effect on the levels of *nifB1*, oxygen decreased expression of *nifB1* about 3- or 4-fold. CnfR1 also activated expression of *nifB1* in an *ntcA* mutant (BP7142) (Fig. 3A), which lacks this key regulatory protein in nitrogen metabolism that is required for heterocyst differentiation (47). Thus, CnfR1 can activate *nifB1* in vegetative cells under aerobic or anaerobic conditions in the presence of fixed nitrogen, and this activation does not require NtcA.

In contrast, expression of CnfR1 at 4 h after Co^2+^ induction in vegetative cells grown with or without fixed nitrogen and with or without oxygen resulted in activation of *nifB2* only in cells starved for fixed nitrogen, while oxygen had relatively little effect on the expression of *nifB2* (Fig. 3B). CnfR1 failed to activate the expression of *nifB2* in an *ntcA* mutant (BP7142), indicating that NtcA was required for activation of *nifB2* by CnfR1 and that repression of *ntcA* expression by fixed nitrogen prevented *nifB2* activation in cells grown with fixed nitrogen.

The experiments described above demonstrated that CnfR2 could not activate *nifB1* in heterocysts or vegetative cells (BP870, Fig. 1A; BP921, Fig. 1B), and this was confirmed by the lack of expression of *nifB1* in Co^2+^-induced cells expressing CnfR2 from the *coaT* promoter in BP1143 (Fig. 3C). However, 4 h after Co^2+^ induction, expression of CnfR2 in cells grown with or without fixed nitrogen resulted in strong activation of *nifB2* only in anaerobic cells (Fig. 3D). In anaerobic cells, fixed nitrogen decreased *nifB2* expression about 5-fold. While NtcA is required for the expression of *cnfR2* (33), CnfR2 made under the control of the *coaT* promoter in an *ntcA* mutant activated the expression of *nifB2*, although expression was decreased severalfold compared to the *ntcA*^+^ strain. Although NtcA was not required for *nifB2* expression in anaerobic cells grown without fixed nitrogen, there was no nitrogenase activity (Fig. 3D; Table 1).

**TABLE 1:** Nif2 nitrogenase activity in *ntcA*, *hetR*, or *nrrA* mutants

Strain	Mutation^*a*^	Growth conditions	Nitrogenase: nmoles ethylene OD_720_^−1^ min^−1^
BP1143	WT	−O_2_ −N, 4 h	0.96 ± 0.05
	WT	−O_2_ +N, 4 h	<0.01
BP7143	*ntcA*	−O_2_ −N, 4 h	<0.01
	*ntcA*	−O_2_ +N, 4 h	<0.01
BP1107	*hetR*	−O_2_ −N, 6 h	0.69 ± 0.03
	*hetR*	−O_2_ +N, 6 h	0.01 ± 0.002
BP1108	*nrrA*	−O_2_ −N, 6 h	0.61 ± 0.04
	*nrrA*	−O_2_ +N, 6 h	<0.01

a WT, wild-type.

### Nif2 nitrogenase activity in *hetR* or *nrrA* mutants.

Several regulatory proteins regulate the response to starvation for fixed nitrogen, including NtcA, NrrA, and HetR. We showed that a *ntcA* mutant reduced the expression of *nifB2* by CnfR2 but did not abolish expression; however, the mutant did not fix nitrogen (Fig. 3D; Table 1). To determine whether NrrA or HetR affected Nif2 expression, we constructed mutants lacking these genes. The *hetR* mutant (BP1107), starved for fixed nitrogen for 6 h under anaerobic conditions, fixed nitrogen well compared to cells grown with fixed nitrogen (Table 1). The *nrrA* mutant (BP1108) gave similar results (Table 1). Hence, neither NrrA nor HetR is essential for the expression of the Nif2 system.

**FIG 4:**
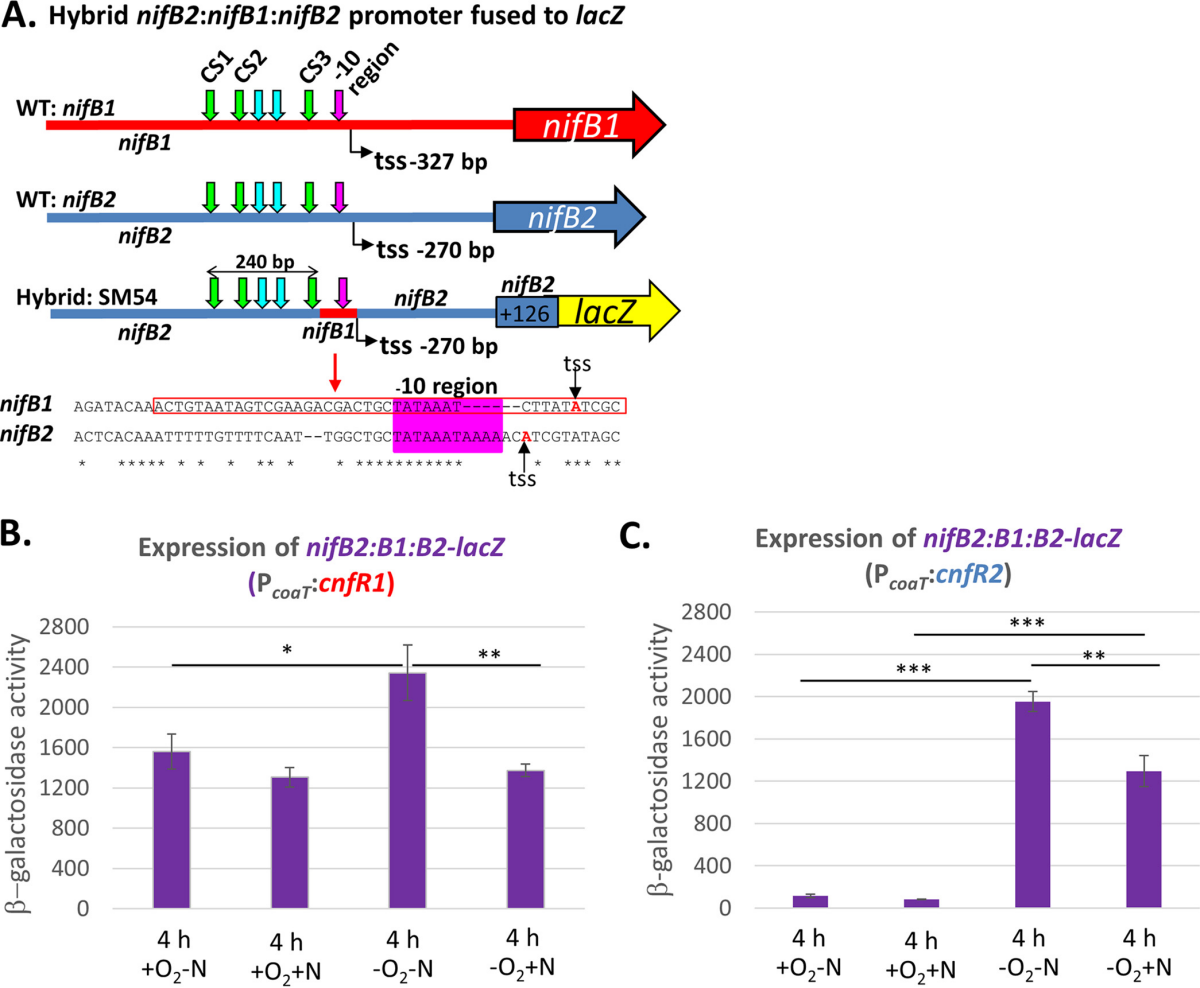
Expression of a hybrid *nifB2*::*nifB1*::*nifB2* promoter by CnfR1 or CnfR2 in vegetative cells. (A) Organization of the SM54 hybrid *nifB2*::*nifB1*::*nifB2* promoter fused to *lacZ.* CS1, CS2, and CS3 are highly conserved regions. The *nifB1* sequence in the red box with the −10 region is indicated by the red portion of SM54; tss, transcription start site. (B, C) Expression of a hybrid *nifB2*::*nifB1*::*nifB2* promoter fused to *lacZ* by CnfR1 in strain BP2197 (B) or CnfR2 in strain BP3197 (C) was determined by β-galactosidase assays in cells grown for 4 h under ±N ±O_2_ conditions. The P*_coaT_* promoter was induced by the addition of Co^2+^ for 4 h before β-galactosidase assays. Statistical analysis: ***, *P < *0.001; **, *P < *0.01; *, *P < *0.05. Nonsignificant comparative values (*P* ≥ 0.05) are not labeled. The horizontal bars below the *P* values provide statistical comparisons for the means of the two values immediately below the ends of the bar and do not include values between these two.

### Expression of a hybrid *nifB2*::*nifB1*::*nifB2* promoter by CnfR1 or CnfR2 in vegetative cells.

The *nifB1* and *nifB2* promoter regions share several highly conserved regions, CS1, CS2, and CS3 (Fig. 4A) (22, 35). We have shown that a hybrid *nifB2*::*nifB1*::*nifB2* promoter that is mostly *nifB2* but has a short *nifB1*-specific region near the transcription start site (SM54) (Fig. 4A) can be activated by CnfR1 in heterocysts and by CnfR2 in anaerobic vegetative cells (35). Here, we determined whether oxygen or fixed nitrogen affected the activation of the *nifB2*::*nifB1*::*nifB2* hybrid promoter driving the expression of *lacZ* in vegetative cells by CnfR1 or CnfR2 made from the Co^2+^-inducible *coaT* promoter. The effects of oxygen or fixed nitrogen on the pattern of expression of the *nifB2*::*nifB1*::*nifB2* promoter were very similar to *nifB1* or *nifB2*. CnfR1 activated the *nifB2*::*nifB1*::*nifB2* promoter in vegetative cells with or without fixed nitrogen or oxygen; however, the highest levels of expression of *lacZ* were in vegetative cells grown anaerobically without fixed nitrogen (Fig. 4B). This pattern is similar to the activation of *nifB1* by CnfR1 made under the control of the *coaT* promoter under the same conditions (Fig. 3A) and different from the pattern of activation of *nifB2* by CnfR1 made under the control of the *coaT* promoter (Fig. 3B). Even though the hybrid promoter is mostly *nifB2*, its activation by CnfR1 in vegetative cells did not show the requirement for NtcA that was evident for activation of *nifB2* by CnfR1 (Fig. 3B). CnfR1 activation of the hybrid promoter was not affected by fixed nitrogen, whereas CnfR1 activation of the *nifB2* promoter was affected by nitrogen. The only difference between the two promoters is the 50-bp *nifB1* region near the transcription start site.

CnfR2 activated the *nifB2*::*nifB1*::*nifB2* promoter in vegetative cells with or without fixed nitrogen but failed to activate the promoter in the presence of oxygen (Fig. 4C); however, the highest levels of expression were for cells grown without oxygen and fixed nitrogen (Fig. 4C). This pattern for activation of the *nifB2*::*nifB1*::*nifB2* promoter is similar to the activation of *nifB2* by CnfR2 made under the control of the *coaT* promoter under the same conditions (Fig. 3D), which is not surprising since most of the promoter is *nifB2*.

### Expression of the Nif2 nitrogenase from the hybrid *nifB2*::*nifB1*::*nifB2* promoter in heterocysts.

**FIG 5:**
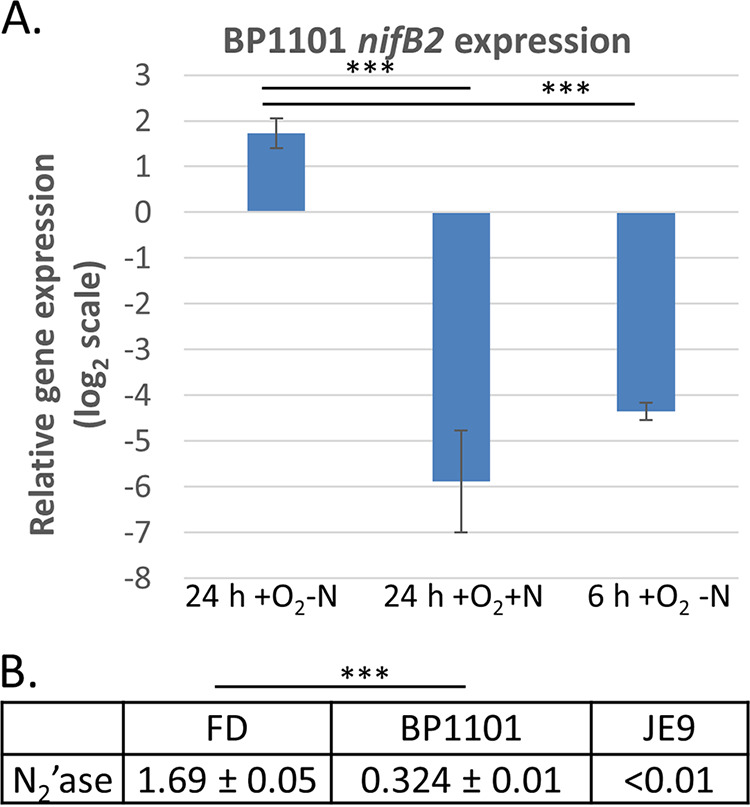
Expression of *nifB2* and the Nif2 nitrogenase in heterocysts. (A) Expression in strain BP1101 of *nifB2* under the control of the hybrid *nifB2*::*nifB1*::*nifB2* promoter was determined by RT-qPCR in cells grown for 24 h under −N +O_2_ conditions (with heterocysts), 24 h under +N +O_2_ conditions (no heterocysts), or 6 h under −N +O_2_ conditions (no heterocysts). Expression of *nifB2* was normalized to *rnpB*, and the values on the *y* axis represent the relative log_2_ fold differences in expression. (B) Nitrogenase activity (N_2_'ase), expressed as nmoles of ethylene OD_720_^−1 ^min^−1^, was determined for wild-type strain FD, JE9 (*nifDKE1* deletion strain), and BP1101 grown for 24 h under −N +O_2_ conditions. Statistical analysis: ***, *P < *0.001; **, *P < *0.01. The horizontal bars below the *P* values provide statistical comparisons for the means of the two values immediately below the ends of the bar and do not include values between these two.

The Nif1 nitrogenase functions well in heterocysts; therefore, it was difficult to determine whether the Nif2 nitrogenase could function in heterocysts unless the Nif1 nitrogenase was absent. Nif2 expression in heterocysts could not be determined using the native *nifB2* promoter, which is not expressed in heterocysts, but could be determined if *nifB2* was expressed under the control of the *nifB2*::*nifB1*::*nifB2* hybrid promoter. In strain BP1101, *nifB2* is driven by the *nifB2*::*nifB1*::*nifB2* hybrid promoter in JE9, a strain with a large deletion of *nifDKE1*, eliminating the Nif1 nitrogenase. While neither CnfR1 nor CnfR2 expressed in heterocysts 24 h after nitrogen step-down induced the expression of *nifB2* from its native promoter (see BP910 and BP870 in Fig. 1A), CnfR1 made in heterocysts from its own promoter activated *nifB2* expression from the *nifB2*::*nifB1*::*nifB2* hybrid promoter. In the absence of heterocysts (24 h +O_2_ +N or 6 h +O_2_ −N), there was no expression of *nifB2* from the hybrid promoter (Fig. 5A). While active Nif2 nitrogenase was synthesized in heterocysts of BP1101, nitrogenase activity was significantly lower in BP1101 than in wild-type strain FD (Fig. 5B).

### CnfR1 activation of the *nifZ2* gene.

**FIG 6:**
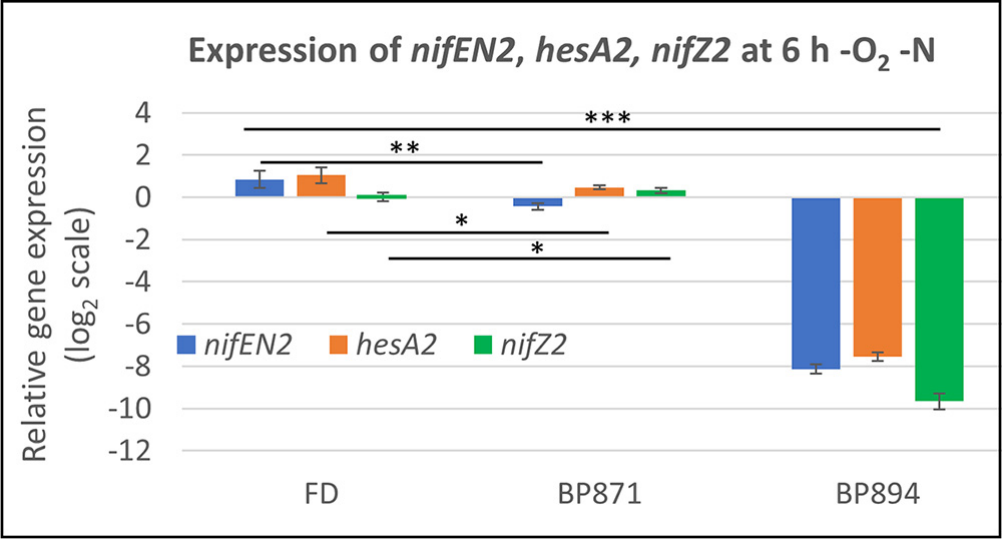
Expression of *nifEN2*, *hesA2*, *and nifZ2.* Expression of *nifEN2*, *hesA2*, and *nifZ2* by CnfR2 (FD), by CnfR1 expressed from the *cnfR2* promoter (BP871), and with neither CnfR1 nor CnfR2 (BP894) was determined by RT-qPCR in cells grown for 6 h under −N −O_2_ conditions. Expression of *nifEN2*, *hesA2*, and *nifZ2* was normalized to *rnpB*, and the values on the *y* axis represent the relative log_2_ fold differences in expression. Statistical analysis: ***, *P < *0.001; **, *P < *0.01; *, *P < *0.05. The *P* values for data for BP894 compared to the other two strains were <0.001. The horizontal bars below the *P* values provide statistical comparisons for the means of the two values immediately below the ends of the bar and do not include values between these two.

We showed previously that genes far downstream of *nifB2*, including *nifEN2* (a fused gene) and *hesA2*, were not expressed in a *cnfR2* mutant, suggesting that they are under the control of the *nifB2* promoter (33). The *nifZT2* genes, which are divergently transcribed from *nifB2*, were also not expressed in a *cnfR2* mutant. Since expression of CnfR1 from the *cnfR2* promoter (BP871) in anaerobic vegetative cells at 6 h after nitrogen step-down induced strong expression of *nifB2* (Fig. 1B), we measured CnfR1-induced expression of *nifEN2*, *hesA2*, and *nifZ2*. Expression of all three genes was activated by CnfR1 in anaerobic vegetative cells nearly as strongly as by CnfR2 in the wild-type strain FD (Fig. 6). Since *nifZ2* is divergently transcribed from *nifB2*, it must have its own promoter, which is activated by CnfR1 as effectively as is the *nifB2* promoter. Thus, all the *nif2* genes can be activated by CnfR2 or CnfR1 (if expressed) in anaerobic vegetative cells.

### Nif2 nitrogenase expression in *Nostoc* sp. PCC 7120.

**FIG 7:**
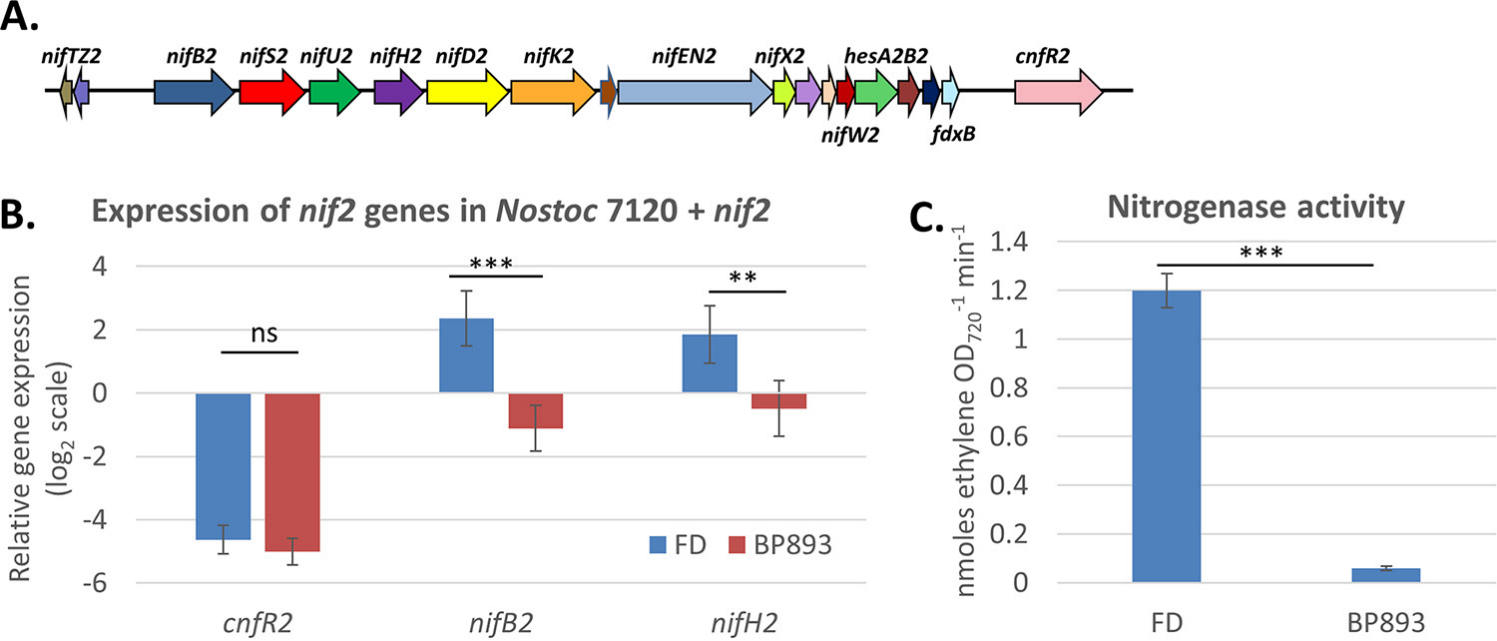
Expression of *nif2* genes and nitrogenase activity in *Nostoc* sp. PCC 7120. (A) The *nif2* genes in BP893. (B) Expression of *cnfR2*, *nifB2*, and *nifH2* in wild-type strain FD and in BP893, a strain of *Nostoc* sp. PCC 7120 with the large *nif2* cluster integrated in the chromosome, was determined by RT-qPCR in cells grown for 6 h under −N −O_2_ conditions. Expression of *cnfR2*, *nifB2*, and *nifH2* was normalized to *rnpB*, and the values on the *y* axis represent the relative log_2_ fold differences in expression. (C) Nitrogenase activity was determined for wild-type strain FD and BP893 grown for 6 h under −N −O_2_ conditions. Statistical analysis: ***, *P < *0.001; **, *P < *0.01; ns, *P > *0.05. The horizontal bars below the *P* values provide statistical comparisons for the means of the two values immediately below the ends of the bar and do not include values between these two.

We have shown previously that CnfR2 expressed from a plasmid in *Nostoc* sp. PCC 7120 activated expression of the *nifB2* promoter in anaerobic vegetative cells (33). Using a fosmid from a genomic library for *A. variabilis* that has the entire *nif2* cluster (Fig. 7A), we constructed a mobilizable plasmid with this *nif2* cluster, which was integrated into the chromosome of *Nostoc* sp. PCC 7120, creating strain BP893. In anaerobic vegetative cells, BP893 expressed *cnfR2* at nearly the same levels as the *A. variabilis* FD control strain; however, expression of *nifB2* and *nifH2* was reduced significantly in BP893 (Fig. 7B). Nitrogenase activity, which was absent in anaerobic vegetative cells of the *Nostoc* sp. PCC 7120 parent strain (data not shown), was present in BP893; however, activity was reduced to about 5% of the activity of the *A. variabilis* FD control strain (Fig. 7C).

## DISCUSSION

CnfR proteins, which have an N-terminal Fe-S-binding domain and a C-terminal HTH domain, are present in all sequenced nitrogen-fixing cyanobacteria, and strains with two Mo-nitrogenase gene clusters have two copies of *cnfR* (20, 21, 33). In *A. variabilis*, the CnfR1 and CnfR2 proteins that control expression of *nifB1* and *nifB2*, respectively, are more similar to each other than to the CnfR proteins of most nonheterocystous cyanobacteria. CnfR2, made only in anaerobic vegetative cells, cannot activate *nifB1* (33); however, when CnfR2 was expressed in heterocysts under the control of the *cnfR1* promoter, it did not activate expression of *nifB1* or *nifB2* (Fig. 1A). This suggests that a factor present in anaerobic vegetative cells, but not in heterocysts, is essential for CnfR2 activation of *nifB2*. In contrast, when CnfR1, which is normally expressed only in heterocysts, was made in anaerobic vegetative cells under the control of the *cnfR2* promoter, it activated expression of both *nifB1* and *nifB2* (Fig. 1B). Therefore, CnfR1 requires no additional heterocyst-specific factor for activation of *nifB1* expression, whereas CnfR2 does require additional factor(s) to activate *nifB2* expression in anaerobic vegetative cells.

It seemed likely that differences in the HTH DNA-binding domains of CnfR1 and CnfR2 specified their binding to the *nifB1* and *nifB2* promoters, respectively. However, exchanging these HTH domains had little effect on the ability of either protein to activate its noncognate promoter when expressed in either heterocysts or in anaerobic vegetative cells (Fig. 1A and B). However, CnfR1-HTH_CnfR2_, expressed in anaerobic vegetative cells under the control of the *cnfR2* promoter, increased expression of *nifB1* and *nifB2* compared to CnfR1, suggesting that the HTH domain of CnfR2 binds better to both promoters, perhaps because the HTH domain interacts with another transcription factor unique to anaerobic vegetative cells. Therefore, while the DNA-binding domains of CnfR1 versus CnfR2 do not provide specificity for activation of their cognate promoters, they do impact the efficacy of activation. Other CnfR1-CnfR2 hybrid proteins that were mostly CnfR2 did not induce *nifB1* expression in heterocysts, but hybrids that included longer regions of the N-terminal half of CnfR1 marginally increased *nifB1* expression compared with those with a shorter N-terminal region from CnfR1 (Fig. 1C). While none of the CnfR1-CnfR2 hybrid proteins activated expression of *nifB1* or *nifB2* in heterocysts well, the N-terminal half of CnfR1 plays a role in determining the specificity of activation of *nifB1* versus *nifB2*.

Exchanging promoters allowed us to determine whether CnfR1 and CnfR2 functioned in anaerobic vegetative cells and in heterocysts, respectively. However, to determine the role of environmental factors, including fixed nitrogen and oxygen, in the activation of the *nifB1* and *nifB2* promoters, we needed to control the expression of *cnfR1* or *cnfR2*. Expression of *cnfR1* or *cnfR2* from the Co^2+^-inducible *coaT* promoter (46, 48) led to a 20- to 50-fold induction by Co^2+^ (Fig. 2C). Co^2+^-induced expression of CnfR1 from the *coaT* promoter activated the *nifB1* promoter in vegetative cells, and expression was somewhat sensitive to oxygen but insensitive to fixed nitrogen (Fig. 3A). In contrast, CnfR1 activated the *nifB2* promoter only in cells starved for fixed nitrogen, but oxygen had little effect (Fig. 3B).

CnfR2 expressed from the *coaT* promoter activated the *nifB2* promoter strongly only in anaerobic vegetative cells but did not activate *nifB1* in heterocysts or aerobic vegetative cells (Fig. 3C and D). Although the *nif2* genes were expressed in anaerobic vegetative cells with fixed nitrogen, nitrogenase activity was poor (Fig. 3D), which suggests that other proteins absent in nitrogen-replete cells (e.g., NtcA) support nitrogenase activity, and/or posttranslational modifications inhibited nitrogenase activity, as has been shown for the Nif1 nitrogenase (49, 50). Oxygen inhibited CnfR2 activation of *nifB2* expression (Fig. 3D). Since *nifB2* transcripts were not affected by oxygen in cells expressing CnfR1, oxygen either inhibits CnfR2 directly, or another transcription factor made only in anaerobic vegetative cells is required for *nifB2* expression. While CnfR2 may be inactivated by oxygen, the fact that CnfR2 cannot activate expression of *nifB2* in heterocysts, which have a low oxygen tension, suggests that a factor induced in vegetative cells by anaerobiosis might be required for CnfR2 activation of *nifB2*.

NtcA is a key regulatory protein in nitrogen metabolism that is required for heterocyst differentiation (47). Therefore, neither *cnfR1* nor *nifB1* is normally expressed in an *ntcA* mutant because it has no heterocysts. Using the *coaT* promoter, we expressed CnfR1 in an *ntcA* mutant and found that it activated the expression of *nifB1* but not *nifB2* in anaerobic vegetative cells (Fig. 3A and B). This is consistent with the fact that *nifB2* was not expressed in cells grown with fixed nitrogen, which represses expression of NtcA (47). NtcA is required for expression of *cnfR2* (33); however, CnfR2 made under the control of the *coaT* promoter in an *ntcA* mutant activated strong expression of *nifB2* (Fig. 3D), but there was no nitrogenase activity. Thus, NtcA is important for the Nif1 nitrogenase because it is required for heterocyst formation, and *cnfR1* and *nifB1* are expressed only in heterocysts. In contrast, NtcA has a more direct role in the Nif2 system since it is required for expression of *cnfR2* and for active nitrogenase, but it is not essential for transcription of *nifB2*.

We were interested in the role of the *nifB1* and *nifB2* promoters in cell-type-specific expression of *nif1* versus *nif2*. The *nifB1* and *nifB2* promoter regions share several highly conserved regions, CS1, CS2, and CS3 (22, 35). We previously described a hybrid *nifB2*::*nifB1*::*nifB2* promoter that is primarily *nifB2*, but has a short region of *nifB1* around the transcription start site (Fig. 4A) that can be activated by CnfR1 in heterocysts and by CnfR2 in anaerobic vegetative cells (35). When the hybrid promoter was activated by CnfR1 or CnfR2 made in all cells from the *coaT* promoter, expression levels for the hybrid promoter were similar to those for *nifB1* or *nifB2*, respectively (Fig. 4B and C). However, unlike the *nifB2* promoter, activation of the hybrid promoter by CnfR1 in vegetative cells did not require NtcA. Similarly, unlike the *nifB2* promoter, CnfR1 activation of the hybrid promoter was not affected by fixed nitrogen, which decreases NtcA expression. Thus, the NtcA requirement for activation of *nifB2* by CnfR1 in vegetative cells is associated with the *nifB2* region around the transcription start site, which is missing in the hybrid promoter. CnfR2 activation of the *nifB2*::*nifB1*::*nifB2* promoter, like the *nifB2* promoter, occurred only in anaerobic vegetative cells, with or without fixed nitrogen, although fixed nitrogen decreased transcription of both promoters. This implies that the transcription start site region of *nifB2* (missing in the hybrid promoter) is not involved in the requirement for anaerobic conditions for activation by CnfR2 but is required for NtcA-dependent nitrogen control of *nifB2* activation by CnfR1.

Although the Nif2 nitrogenase normally functions in anoxic vegetative cells, the *nifB2*::*nifB1*::*nifB2* hybrid promoter provided a means to determine whether the Nif2 nitrogenase could function in the microoxic environment of the heterocyst. In a *nifDK1*-deletion strain, we found that expression of the *nif2* genes, under the control of the hybrid promoter, was activated by CnfR1 in heterocysts and produced active nitrogenase (Fig. 5). The *nif2* cluster that was expressed from the hybrid promoter has *nifB2*, *nifS2*, *nifU2*, *nifH2*, *nifD2*, *nifK2*, *nifEN2*, *nifX2*, *nifW2*, *hesA2*, and *hesB2* as well as several conserved *nif2* genes of unknown function. However, the *nif2* cluster lacks *nifZ2* and *nifT2*, which are transcribed divergently from *nifB2* (19) and lack the hybrid promoter, which is separated from *nifZT2* by the integrated plasmid. The *nifV*, *nifZ1*, and *nifT1* genes are distant from the primary *nif1* cluster (19, 51), so their expression would be unaffected by the *nifDK1* deletion, and NifV, NifZ1, and NifT1 likely functioned to support Nif2 nitrogenase synthesis in heterocysts, although possibly not as well as the Nif2 proteins.

Since the region between the two divergent *nif2* operons (*nifZT2* and the large cluster that begins with *nifB2*) has only one apparent binding site for CnfR proteins (20, 22, 35), CnfR2 controls expression of all the *nif2* genes using this intergenic region. When expressed in anaerobic vegetative cells, CnfR1 (and CnfR2) can control the expression of all *nif2* genes using the single regulatory region between *nifZ2* and *nifB2*. (Fig. 6).

Transferring the genes to fix nitrogen to nonnitrogen-fixing strains is an important first step in the long-term goal of creating nitrogen-fixing plants. However, many genes, some unknown, are required for the synthesis of a functional nitrogenase. The compact *nif2* cluster of *A. variabilis* has all the *nif* genes and *cnfR2*. We transferred the *nif2* cluster to *Nostoc* sp. PCC 7120 where Nif2 nitrogenase was made, allowing the strain to fix nitrogen in anaerobic vegetative cells (Fig. 7). Similarly, a large chromosomal region with 25 *nif* and *nif*-related genes from the cyanobacterium L. boryana was integrated into the genome of the unicellular cyanobacterium *Synechocystis* 6803, which has no nitrogenase genes (52). We saw about 5% of the normal *A. variabilis* nitrogenase activity in vegetative cells for the *Nostoc* sp. PCC 7120 strain with the *nif2* genes, which was much more than the 0.26% nitrogenase reported for *Synechocystis* 6803 compared to L. boryana, the source of the *nif* genes (52). It is apparent that even among cyanobacteria, the ability to express cyanobacterial *nif* genes and make a functional nitrogenase depends on unknown factors that are important for optimal function.

## MATERIALS AND METHODS

### Construction of hybrid *cnfR1*/*cnfR2* strains.

Hybrid *cnfR1*/*cnfR2* strains were constructed by integration of plasmids into the chromosome of BP894 (*cnfR1*, *cnfR2* double deletion mutant). The details of the construction of all plasmids are included in Table S2 in the supplemental material, and primers used in the construction are listed in Table S3. Plasmids pBP870-pBP873, pBP907, pBP910, pBP920, pBP921, pBP950, pBP951, and pBP952 were conjugated into BP894 (*cnfR1*, *cnfR2* double deletion mutant), as described previously (53), where they integrated by single recombination into the *cnfR1* or *cnfR2* promoter regions to create strains BP870, BP871, BP872, BP873, BP907, BP910, BP920, BP921, BP950, and BP951. The integration of the plasmids was verified to be a promoter cross by PCR.

### Construction of Co^2+^-inducible strains.

Co^2+^-inducible *lacZ*, *cnfR1*, or *cnfR2* genes in plasmids pBP1141, pBP1142, and pBP1143 were constructed by fusion PCR, as described in Table S2 in the supplemental material. Plasmid pBP1141 was conjugated into FD, and plasmids pBP1142 and pBP1143 were conjugated into both BP894 and MM3, an *ntcA* mutant (34), where they integrated by single recombination into the *frtA* region to create strains BP1141 (P*_coaT_*::*lacZ* in FD), BP1142 (P*_coaT_*::*cnfR1* in BP894), BP1143 (P*_coaT_*::*cnfR2* in BP894), BP7142 (P*_coaT_*::*cnfR1* in MM3), and BP7143 (P*_coaT_*::*cnfR2* in MM3). The integration of the plasmids in *frtA* was verified by PCR.

### Construction of hybrid *nifB* promoter strains.

Since cyanobacterial strains BP1142 and BP1143 were already Nm^r^ and had the P*_coaT_*::fusions integrated into the *frtA* region, a different antibiotic reporter and integration region was required to insert the hybrid *pnifB2*::*nifB1*::*nifB2* promoter into these strains. Plasmid pBP1195 was engineered as described in Table S2 to be Em^r^ and to allow integration into the *modA* region. The *pnifB2*::*nifB2*::*nifB2* promoter was inserted into pBP1195, as described in Table S2, to create pBP1197. Plasmid pBP1197 was conjugated into BP1142 and BP1143 where it integrated via single recombination into the *modA* region to create cyanobacterial strains BP2197 and BP3197, respectively. The integration of the plasmid in the *modA* gene was verified by PCR. The hybrid *pnifB2*::*nifB1*::*nifB2* promoter was extended about 870 bp into the *nifB2* gene, as described in Table S2, to create plasmid pBP1101. Plasmid pBP1101 was conjugated into JE9 (*xisA-nifE1* deletion mutant [54]), where it integrated by single recombination in the *nifB2* gene to drive expression of the *nifBSUHDKENXW2-hesAB2* operon, thereby creating BP1101. The integration of the hybrid promoter upstream of the structural *nif2* genes was verified by PCR.

### Construction of *hetR* and *nrrA* mutants.

Plasmids pBP1107 and pBP1108 were constructed as described in Table S2 to delete the *hetR* and *nrrA* genes, respectively. Plasmids pBP1107 and pBP1108 were conjugated into strain FD where they integrated by single recombination into the *hetR* or *nrrA* regions. Single recombinants were selected by Em^r^, followed by *sacB* selection on 10% sucrose plates for double recombinants (55, 56) to create cyanobacterial strains BP1107 and BP1108. PCR was used to verify that there were no wild-type copies of the genes.

### Construction of *Nostoc* sp. PCC 7120 with the *nif2* cluster.

We created plasmid pBP716 by inserting a 1.9-kb SmaI fragment containing the *aadA* cassette (Sp^r^Sm^r^) from pRL5801 into the SmaI site of pRL2948a. The Escherichia coli strain containing pAAWZ1787 (a fosmid from the Joint Genome Institute [JGI] containing the *nifB2* cluster from genes ava_4241–ava_4266) was electroporated with pKM208. pKM208 contains all the genes necessary for recombineering under the P*tac* promoter; *red* and *gam* genes are repressed by *lacI* (57). The plasmid also has a temperature-sensitive origin of replication, which allows the plasmid to be cured from the strain by growth at 37°C. A 2.1-kb PCR product generated from pBP716 using primers FosOriTSp-L2 and FosOriTSp-R2 containing the *oriT* site (required for conjugation of the plasmid to cyanobacteria) and the Sp^r^Sm^r^ cassette was treated with DpnI to remove plasmid DNA and recombineered into pAAWZ1787 at the Cm^r^ cassette using pKM208 by electroporation. The E. coli strain containing pAAWZ1787 and pKM208 was treated with isopropyl-β-d-thiogalactopyranoside (IPTG) just before electroporation to activate the recombination genes. The recombineered plasmid, pBP890, containing the *nif2* gene cluster in addition to *oriT* and the Sp^r^Sm^r^ cassette was screened for replacement of the Cm^r^ cassette with the Sp^r^Sm^r^ cassette. Plasmid pBP890 was electroporated into HB101 pRL528 and then conjugated into BP291 (a *Nostoc* sp. PCC 7120 strain containing the *frtRABC* operon that allows the strain to use fructose as a carbon source [58]), as described previously (53), creating strain BP893. The presence of the *nif2* region in BP893 was verified by PCR.

### Aerobic nitrogen step-down experiments.

Aerobic cultures were grown for about 10 generations in an 8-fold dilution of AA medium (AA/8) (59) with 5 mM NH_4_Cl and 10 mM *N*-tris(hydroxymethyl)methyl-2-aminoethanesulfonic acid (TES), pH 7.2, and then diluted 1:100 in the same medium and grown to an optical density at 720 nm (OD_720_) of 0.1 to 0.2. Cultures were washed and diluted with AA/8 to an OD_720_ of 0.1 to achieve nitrogen step-down. At least three 25-ml biological replicates of each culture were grown for 24 h at 30°C with shaking and illumination at 80 to 100 μE m^−2^ s^−1^ in 125-ml flasks.

### Anaerobic nitrogen step-down experiments.

For anaerobic nitrogen step-down experiments, cells were grown in air in the light with shaking in AA/8 with 5.0 mM fructose, 5.0 mM NH_4_Cl, and 10 mM TES, pH 7.2, to an OD_720_ of 0.5 to 0.6. Cells were washed with AA/8 and resuspended in AA/8 with 10 mM fructose and 10 μM dichlorophenyldimethylurea (DCMU; to inhibit oxygen evolution from photosystem II) to an OD_720_ of 0.6. At least three, 8-ml biological replicates were aliquoted into 16-ml Hungate tubes for each condition tested; 5 mM NH_4_Cl and 10 mM TES, pH 7.2, (+N condition) were added to the samples, when applicable, and samples were flushed with dinitrogen for 3 min. The cultures were then incubated for 4 to 6 h anaerobically at 30°C with illumination at 80 to 100 μE m^−2^ s^−1^, and induction of nitrogenase activity was verified by acetylene reduction assays.

### Expression of genes from the *coaT* promoter.

The *cnfR* genes under the control of the *coaT* promoter were grown in Co^2+^-free BG-11^o^ (60) medium containing 5 mM NH_4_Cl and 10 mM TES, pH 7.2, 5 μg ml^−1^ neomycin, and 10 μg l^−1^ cobalamin (vitamin B_12_) (because cobalt is required for cobalamin synthesis) and then diluted 1:100 in conditioned medium (medium further depleted of cobalt by prior growth of strain FD in Co^2+^-free BG-11^0^ medium lacking fixed N) and grown to an OD_720_ of 0.1 to 0.2. Cells were washed free of fixed nitrogen and diluted with conditioned Co^2+^-free BG-11^0^ containing 10 μg l^−1^ cobalamin and lacking fixed nitrogen to an OD_720_ of 0.1. At least three 25-ml biological replicates of each culture were grown for 20 h at 30°C with shaking and illumination at 80 to 100 μE m^−2^ s^−1^ in 125-ml flasks. After confirmation of heterocyst formation at 20 h after nitrogen step-down, 2 μM CoCl_2_ was added to some cultures for 4 h to induce the *cnfR* genes driven by the *coaT* promoter. AA/8 medium could not be used for induction of the *coaT* promoter because the EDTA in the medium caused chelation of the Co^2+^.

For anaerobic nitrogen step-down experiments, cells were grown aerobically, as described above, in Co^2+^-free BG-11^0^ medium containing 5 mM NH_4_Cl and 10 mM TES, pH 7.2, 5 mM fructose, 5 μg ml^−1^ neomycin, and 10 μg l^−1^ cobalamin to an OD_720_ of 0.5 to 0.6. Cultures were diluted 1:100 in the same medium and grown to an OD_720_ of 0.3 to 0.4. Cells were washed free of nitrogen with conditioned Co^2+^-free BG-11^0^ lacking fixed nitrogen and resuspended in conditioned cobalt-free BG-11^0^ (−N) containing 10 μg ml^−1^ cobalamin, 10 mM fructose, and 10 μM DCMU to an OD_720_ of 0.6. At least three, 8-ml biological replicates were aliquoted into 16-ml Hungate tubes for each condition tested; 5 mM NH_4_Cl and 10 mM TES, pH 7.2 (+N condition), and 2 μM CoCl_2_ (to induce P*_coaT_*) were added to samples, when applicable, and samples were flushed with dinitrogen for 3 min. The cultures were then incubated for 4 h anaerobically at 30°C with illumination at 80 to 100 μE m^−2^ s^−1^, and induction of *nif* genes was verified by acetylene reduction assays.

### Acetylene reduction assays.

Acetylene reduction assays have been described previously (33). For acetylene reduction of anaerobic cultures, 1 ml of acetylene gas was added to Hungate tubes 30 min before the end of the anaerobic incubation period. For aerobic cultures, 1 ml of acetylene gas was added to 5-ml cultures stoppered in 16-ml Hungate tubes and incubated for 1 h at 30°C, with illumination at 80 to 100 μE m^−2^ s^−1^. Samples (250 μl) of headspace gas were removed via a gas-tight needle/syringe and injected into a Shimadzu gas chromatograph equipped with a 6-foot Porapak N column. The column temperature was 75°C.

### β-Galactosidase assays.

β-Galactosidase assays were performed in 96-well microtiter plates using 250 μl of sample for at least 3 biological replicates, with quadruple technical replicates for each biological replicate. β-Galactosidase assays were performed as previously described (35). OD_420_ and OD_665_ measurements were taken to determine the amount of 2-nitrophenol (OD_420_) and to correct for chlorophyll and light scattering from permeabilized cells (OD_665_). Calculations were performed using the following equation, developed empirically: β-galactosidase = 1,000 × ([OD_420_ – (1.58 × OD_665_)]/[OD_720_ × time of assay (in minutes)]). The values from the quadruple technical replicates were averaged for each of the three biological replicates. These three averages for the biological replicates were used to calculate the average, and the standard deviation is shown in the graphs as error bars.

Aerobic cultures of BP1141, BP2197, and BP3197 were grown in Co^2+^-free BG-11 medium and induced as described above for expression of the *coaT* promoter for about 10 generations in Co^2+^-free BG-11 medium containing, 10 μg l^−1^ cobalamin, and antibiotics and then diluted 1:100 in the same medium and grown to an OD_720_ of 0.1 to 0.2. For BP1141, various concentrations of CoCl_2_ (0 to 20 μM) and ZnCl_2_ (0 to 100 μM) were added to four 2-ml biological replicates of BP1141, which were grown for 2 h at 30°C with shaking and illumination at 80 to 100 μE m^−2^ s^−1^ in 12-well plates before the β-galactosidase assays. For BP2197 and BP3197, CoCl_2_ at 2 μM (to induce P*_coaT_*) and 5 mM NH_4_Cl and 10 mM TES, pH 7.2 (+N condition), when applicable, were added to samples. Cultures were grown for 4 h at 30°C with shaking and illumination at 80 to 100 μE m^−2^ s^−1^ before the β-galactosidase assays.

For anaerobic nitrogen step-down experiments, cells of BP2197 and BP3197 were grown in Co^2+^-free BG-11 medium and induced as described above for expression of the *coaT* promoter. At least three, 8-ml biological replicates were aliquoted into 16-ml Hungate tubes for each condition tested; 2 μM CoCl_2_ (to induce P*_coaT_*) and 5 mM NH_4_Cl and 10 mM TES, pH 7.2 (+N condition), when applicable, were added to samples, and samples were flushed with dinitrogen for 3 min and incubated for 4 h at 30°C with illumination at 80 to 100 μE m^−2^ s^−1^ before the β-galactosidase assays.

### RNA isolation and reverse transcriptase-quantitative PCR.

RNA isolation and reverse transcriptase-quantitative PCR (RT-qPCR) were performed as described previously (28, 33, 58). RNA was isolated using TRI Reagent (Sigma) and subjected to DNase digestion (Turbo DNA-free kit; Ambion). A total of 40 ng of RNA was converted to cDNA in a 10-μl reaction mixture using iScript reverse transcription supermix for RT-qPCR (Bio-Rad). The cDNA was diluted 1:20 to 0.2 ng μl^−1^. The qPCRs used 0.8 ng of cDNA in a 10-μl reaction mixture with 5 pmol of gene-specific primers and 1× SsoAdvanced SYBR green supermix (Bio-Rad). All genes were compared to the housekeeping gene *rnpB* to determine Δ*^Cq^* values.

### Statistical analysis.

Data are shown as the mean ± standard deviation. The significance of the differences between the means for two values was analyzed using an unpaired, two-tailed Student’s *t* test, with *P* values of < 0.05 considered statistically significant.

### Data availability.

All processed data (e.g., relative expression and β-galactosidase values) used in the experiments are provided here. Additional raw data may be requested from the authors.
