# Supplementary material for: Cross-Activation of Two Nitrogenase Gene Clusters by CnfR1 or CnfR2 in the Cyanobacterium Anabaena variabilis
Source: Microbiol Spectr. 2021 Oct 6;9(2):e01060-21. doi: 10.1128/Spectrum.01060-21 (PMC8510180; doi:10.1128/Spectrum.01060-21)
Supplement: Supplementary file 1 — Supplemental material. Download Spectrum.01060-21-s0001.pdf, PDF file, 0.6 MB [file spectrum.01060-21-s0001.pdf]

Supplementary Materials

Cell-type-specific activation of two nitrogenase gene clusters by CnfR1 or CnfR2 in the cyanobacterium *Anabaena variabilis*

Brenda S. Pratte and Teresa Thiel*

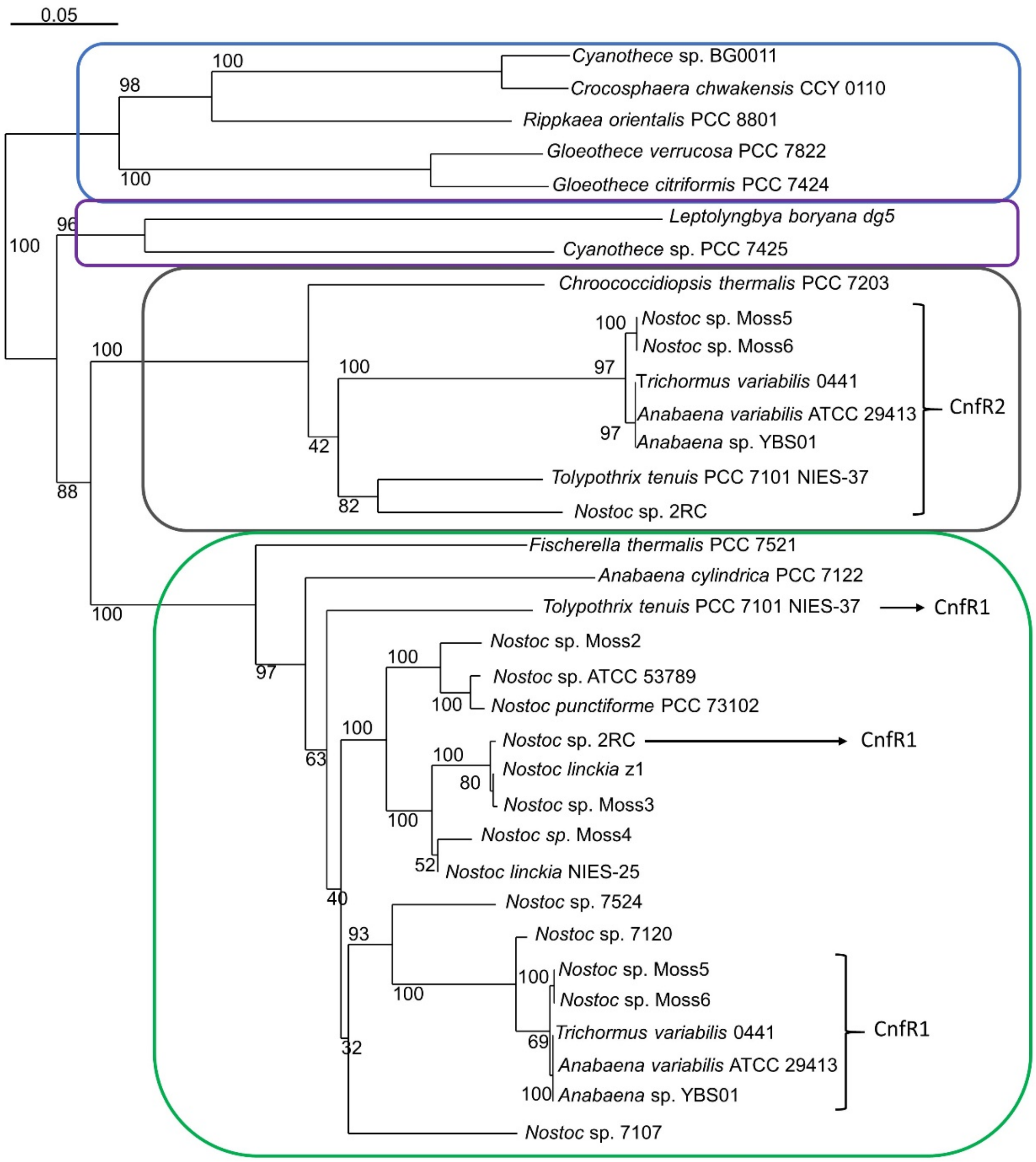

Fig. S1. Phylogenetic tree of CnfR proteins. For strains that have two *nif* systems and thus two CnfR proteins, each copy is labeled on the tree. Blue box = CnfR in unicellular strains, Purple box = other CnfR genes, Grey box = CnfR2 family, Green box = CnfR1 genes in heterocystous cyanobacteria. Accession numbers for the genes are provided in Table S1. This BioNJ distance phylogeny tree was constructed using the program SeaView (1) with 100-replicate bootstrap values.

Table S1: Strains included in Fig. S1.

IMG Gene ID	Genome Name	IMG Locus Tag
637232894	*Nostoc* sp. PCC 7120	all2512
640625288	*Crocosphaera chwakensis* CCY 0110	CY0110_23386
642599653	*Nostoc punctiforme* PCC 73102	Npun_R0334
643475300	*Rippkaea orientalis* PCC 8801	PCC8801_2251
643480389	*Gloeothece citriformis* PCC 7424	PCC7424_2132
643588188	*Cyanothece* sp. PCC 7425	Cyan7425_5231
646565923	*Anabaena variabilis* ATCC 29413 – *cnfR1*	Ava_0444
646569759	*Anabaena variabilis* ATCC 29413 – *cnfR2*	Ava_4260
648188610	*Gloeothece verrucosa* PCC 7822	Cyan7822_3694
2503614847	*Chroococcidiopsis thermalis* PCC 7203	Chro_4567
2503739381	*Nostoc* sp. PCC 7107	Nos7107_0753
2504129697	*Anabaena cylindrica* PCC 7122	Anacy_0077
2509812238	*Nostoc* sp. PCC 7524	Nos7524_4670
2550718290	*Fischerella thermalis* PCC 7521	UYKDRAFT_03084
2631162521	*Nostoc* sp. Moss2	Ga0080672_123114
2631163828	*Nostoc* sp. Moss5 – *cnfR2*	Ga0080674_104223
2631167682	*Nostoc* sp. Moss5– *cnfR1*	Ga0080674_1044084
2631177118	*Nostoc* sp. Moss4	Ga0080676_1211926
2634115732	*Nostoc* sp. Moss3	Ga0080673_0878
2634127375	*Nostoc* sp. Moss6 – *cnfR1*	Ga0080675_0504
2634128697	*Nostoc* sp. Moss6 – *cnfR2*	Ga0080675_1826
2774718376	*Leptolyngbya boryana* dg5	Ga0226171_122313
2776140273	*Tolypothrix tenuis* PCC 7101 NIES-37 – *cnfR2*	Ga0263545_16524
2776145131	*Tolypothrix tenuis* PCC 7101 NIES-37 – *cnfR1*	Ga0263545_165389
2833621481	*Nostoc linckia* NIES-25	Ga0263765_836
2843035298	*Nostoc linckia* z1	Ga0336783_054_19484_21073
2845924250	*Nostoc* sp. ATCC 53789	Ga0336842_155_87608_89203
2883181653	*Trichormus variabilis* 0441 – *cnfR2*	Ga0443053_01_273221_274816
2883182992	*Trichormus variabilis* 0441 – *cnfR1*	Ga0443053_01_1861810_1863441
2883410175	*Anabaena* sp. YBS01 – *cnfR2*	Ga0439566_01_365100_366695
2883411055	*Anabaena* sp. YBS01 – *cnfR1*	Ga0439566_01_1343754_1345343
2886593859	*Cyanothece* sp. BG0011	Ga0452603_049_1738_3336
2909711544	*Nostoc* sp. 2RC – *cnfR1*	Ga0478412_158_79026_80615
2909716834	*Nostoc* sp. 2RC – *cnfR2*	Ga0478412_853_43_1659

IMG: https://img.jgi.doe.gov/

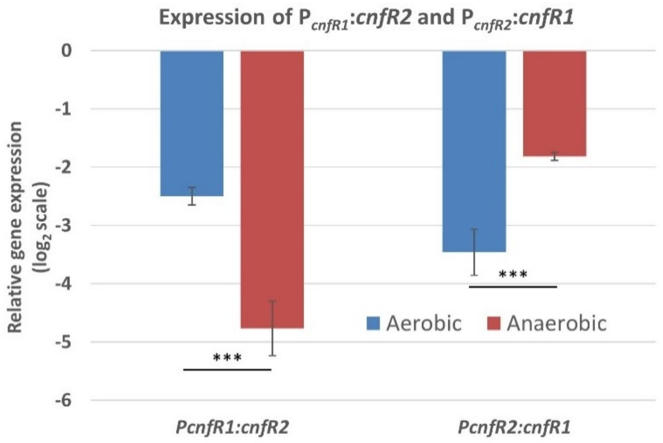

Fig. S2. Expression of *cnfR2* under the control of the *cnfR1* promoter or *cnfR1* under the control of the *cnfR2* promoter. Expression from P_{cnfR1}:*cnfR2* (BP870) and P_{cnfR2}:*cnfR1* (BP871)was determined by RT-qPCR in aerobic cells grown 24 h -N +O_2, leading to formation of heterocysts that activate the *cnfR1* promoter, or anaerobic cells grown 6 h -N -O_2, leading to vegetative cells that activate the *cnfR2* promoter. Expression of *cnfR1* and *cnfR2* was normalized to *rnpB* and the values on the y-axis represents the relative log_2 fold differences in expression Statistical analysis: $P < 0.001$ (***). The horizontal bars below the *P*-values provide statistical comparisons of the means for the two values immediately below the ends of the bar and do not include values between these ends.

Table S2. Strains and plasmids.

Strains	Relevant characteristics	Source or reference
Anabaena variabilis FD	A derivative of *A. variabilis* 29413	(2)
Nostoc sp. PCC 7120	A filamentous nitrogen-fixing cyanobacterium that differentiates heterocysts, but lacks the Nif2 system	
Synechocystis sp. PCC 6803	non-filamentous, non-nitrogen fixing microorganism and source of the Co^{++}-inducible promoter (P*coaT*).	
BP291	*Nostoc sp.* strain PCC 7120 derivative that expresses *frtRABC*.	(3)
BP870	pBP870 (*PcnfR1:cnfR2* fusion) recombined into the *cnfR1 promoter of A. variabilis* BP894 (*ΔcnfR1 ΔcnfR2* strain).	This study
BP871	pBP871 (*PcnfR2:cnfR1* fusion) recombined into the *cnfR2* promoter of *A. variabilis* BP894 (*ΔcnfR1 ΔcnfR2* strain).	This study
BP873	pBP873 (*PcnfR2:cnfR2:cnfR1HTH* fusion) recombined into the *cnfR2* promoter of *A. variabilis* BP894 (*ΔcnfR1 ΔcnfR2* strain).	This study
BP893	pBP890 (*nif2* operon with Sp^r cassette) in BP291 (*Nostoc sp.* strain PCC 7120 containing the *frtRABC* operon)	This study
BP894	*A. variabilis* FD containing a deletion mutation in both *cnfR1* and *cnfR2 (ΔcnfR1 ΔcnfR2)*.	(4)
BP907	pBP907 (*PcnfR2:cnfR1:cnfR2HTH* fusion) recombined into the *cnfR2* promoter of *A. variabilis* BP894 (*ΔcnfR1 ΔcnfR2* strain).	This study
BP910	pBP910 (*PcnfR1:cnfR2:cnfR1HTH* fusion) recombined into the *cnfR1* promoter of *A. variabilis* BP894 (*ΔcnfR1 ΔcnfR2* strain).	This study
BP920	pBP920 (*PcnfR1:cnfR1* fusion) recombined into the *cnfR1* promoter of *A. variabilis* BP894 (*ΔcnfR1 ΔcnfR2* strain).	This study

BP921	pBP921 (*PcnfR2:cnfR2* fusion) recombined into the *cnfR2* promoter of *A. variabilis* BP894 (*ΔcnfR1 ΔcnfR2* strain).	This study
BP950	pBP950 (*PcnfR1:cnfR1:cnfR2:cnfR1HTH* fusion) recombined into the *cnfR1* promoter of *A. variabilis* BP894 (*ΔcnfR1 ΔcnfR2* strain).	This study
BP951	pBP951 (*PcnfR1:cnfR1:cnfR2:cnfR1HTH* fusion) recombined into the *cnfR1* promoter of *A. variabilis* BP894 (*ΔcnfR1 ΔcnfR2* strain).	This study
BP952	pBP952 (*PcnfR1:cnfR1:cnfR2:cnfR1HTH* fusion) recombined into the *cnfR1* promoter of *A. variabilis* BP894 (*ΔcnfR1 ΔcnfR2* strain).	This study
BP1101	*PnifB2:B1:B2* promoter singly recombined to drive the *nifB2* operon in JE9 (a *xisA-nifE1* deletion strain of *A. variabilis).*	This study
BP1107	pBP1107 doubly recombined both upstream and downstream of *hetR* in *A. variabilis* FD to create a *hetR* deletion.	This study
BP1108	pBP1108 doubly recombined both upstream and downstream of *nrrA* in *A. variabilis* FD to create a *nrrA* deletion.	This study
BP1141	pBP1141 (*PcoaT:lacZ* fusion) in *A. variabilis* FD	This study
BP1142	pBP1142 (*PcoaT:cnfR1* fusion) in *A. variabilis* BP894 (*ΔcnfR1ΔcnfR2* strain)	This study
BP1143	pBP1143 (*PcoaT:cnfR2* fusion) in *A. variabilis* BP894 (*ΔcnfR1ΔcnfR2* strain)	This study
BP2197	pBP1197 (*PnifB2:B1:B2:lacZ* fusion) in *A. variabilis* BP1142 (*PcoaT:cnfR1* in *ΔcnfR1ΔcnfR2* strain)	This study
BP3197	pBP1197 (*PnifB2:B1:B2:lacZ* fusion) in *A. variabilis* BP1143 (*PcoaT:cnfR2* in *ΔcnfR1ΔcnfR2* strain)	This study
BP7142	pBP1142 (*PcoaT:cnfR1* fusion) in *A. variabilis* MM3 (*ntcA*$^-$ strain)	This study
BP7143	pBP1143 (*PcoaT:cnfR2* fusion) in *A. variabilis* MM3 (*ntcA*$^-$ strain)	This study

JE9	*xisA-nifEI* region of the *nif1I* genes interrupted with an Nm^r gene	(5)
MM3	*A. variabilis* FD containing a mutation in *ntcA*.	(6)

Plasmids		
pAAWY22574	Plasmid from JGI containing the *nrrA* region	JGI
pAAWY8706	Plasmid from JGI containing the *hetR* region	JGI
pAAWZ1787	Fosmid from JGI containing the *nif2* cluster from *ava_4241-ava_4266.*	JGI
pBP639	Vector with a promoterless *lacZ* for promoter fusions and a Tc^r cassette for selection of promoter fragments; allows for recombination into a gene promoter region of the chromosome; Km^rTc^r	(7)
pBP716	1.9-kb SmaI fragment containing the *aadA* cassette (Sp^rSm^r) from pRL5801 into the SmaI site of pRL2948a, containing *oriT* site required for conjugation into cyanobacteria.	This study
pBP744	Vector with a promoterless *lacZ* for promoter fusions and a Tc^r cassette for selection of promoter fragments; allows for recombination into the *frtBC* region of the chromosome; Km^rTc^r	(4)
pBP870	*PcnfR1:cnfR2* fusion was inserted between the BglII and SacI sites of pBP639.	(4)
pBP871	*PcnfR2:cnfR1* fusion was inserted between the BglII and SacI sites of pBP639.	(4)
pBP872	*PcnfR1:cnfR1cnfR2HTH* fusion was created using fusion PCR; *PcnfR1:cnfR1HTH*- and *cnfR2HTH* fragments were amplified using primers sets patB1L102 + patB1HTHB2-R and patB1HTHB2-L + patB2-R(Fus), respectively, fused into a single fragment, and then inserted between the BglII and SacI sites of pBP639.	This study
pBP873	*PcnfR2*:cnfR2cnfR1HTH fusion was created using fusion PCR; *PcnfR2:cnfR2HTH*- and *cnfR1HTH* fragments were amplified using primer sets patB2-L201 + patB2HTHB1-R and patB2HTHB1-L + patB1-R(Fus), respectively, fused into a single fragment, and then inserted between the BglII and SacI sites of pBP639.	This study

pBP890	2.1-kb PCR fragment, containing the *oriT* site and *aadA* (Sp^rSm^r) cassette, was generated from pBP716 using primers FosOriTSp-L2 + FosOriTSp-R2 and recombineered into pAAWZ1787 at the Cm^r cassette.	This study
pBP907	*PcnfR2:cnfR1cnfR2HTH* fusion was created using fusion PCR; *PcnfR2* and *cnfR1:cnfR2HTH* were amplified from pBP873 using primer set patB2-L201 + P2patB1-R and from pBP872 using primer set P2patB1-L + patB2-R(Fus), respectively, fused into a single fragment, and then inserted between the BglII and SacI sites of pBP639.	This study
pBP910	*PcnfR1:cnfR2cnfR1HTH* fusion was created using fusion PCR; *PcnfR1* and *cnfR2:cnfR1HTH* were amplified from pBP872 using primer set patB1L102 + P1patB2-R and from pBP873 using primer set P1patB2-L + patB1-R(Fus), respectively, fused into a single fragment, and then inserted between the BglII and SacI sites of pBP639.	This study
pBP920	*PcnfR1:cnfR1* (wild-type) was amplified by PCR using primers patB1L102 and patB1-R(Fus) and then inserted between the BglII and SacI sites of pBP639.	This study
pBP921	*PcnfR2:cnfR2* (wild-type) was amplified by PCR using primers patB2L-201 and patB2-R(Fus) and then inserted between the BglII and SacI sites of pBP639.	This study
pBP950	*PcnfR1:cnfR1*$^{1\text{-}179}$ *cnfR2*$^{174\text{-}1425}$*cnfR1HTH*$^{1420\text{-}1590}$ fusion was created using fusion PCR; *pcnfR1:cnfR1*$^{1\text{-}179}$ and *cnfR2*$^{174\text{-}1425}$*cnfR1HTH*$^{1420\text{-}1590}$ fragments were amplified from pBP872 using primer set patB1L102 + P1cnfR2fusion1-R and from pBP873 using primer set P1cnfR2fusion1-L + patB1-R (fus), respectively, fused into a single fragment, digested with BglII and SacI, and then inserted between the BglII and SacI sites of pBP639.	This study
pBP951	*PcnfR1:cnfR1*$^{1\text{-}476}$*cnfR2*$^{486\text{-}1425}$*cnfR1HTH*$^{1420\text{-}1590}$ fusion was created using fusion PCR; *pcnfR1:cnfR1*$^{1\text{-}476}$ and *cnfR2*$^{486\text{-}1425}$*cnfR1HTH*$^{1420\text{-}1590}$ fragments were amplified from pBP872 using primer set patB1L102+ P1cnfR2fusion2-R and from pBP873	This study

	using primer set P1cnfR2fusion2-L + patB1-R (fus), respectively, fused into a single fragment, digested with BglII and SacI, and then inserted between the BglII and SacI sites of pBP639.	
pBP952	*PcnfR1:cnfR1*$^{1-992}$*cnfR2*$^{1002-1425}$*cnfR1HTH*$^{1420-1590}$ fusion was created using fusion PCR; *PcnfR1:cnfR1*$^{1-992}$ and *cnfR2*$^{1002-1425}$*cnfR1HTH*$^{1420-1590}$ fragments were amplified from pBP902 using primer set patB1L102+ P1cnfR2fusion3-R and from pBP903 using primer set P1cnfR2fusion3-L + patB1-R (fus), respectively, fused into a single fragment, digested with BglII and SacI, and then inserted between the BglII and SacI sites of pBP639.	
pBP1101	*PnifB2:nifB1:nifB2* promoter was amplified by PCR from pSM54 using primers pnifB2-L1(JJ) & pnifB2-BsaR1 and an additional 870-bp region at the 5' end of the nifB2 gene was amplified from FD by PCR using primers pnifB2-BsaL1 & nifB2-SmaR2 to create a larger region for recombination for the hybrid promoter. These fragments were digested with BglII & BsaI and BsaI & SmaI, respectively, and inserted between the BglII and SmaI sites of pBP639.	This study
pBP1104	A *hetR* deletion was created using fusion PCR; fragments upstream and downstream of *hetR* were amplified from pAAWY8706 using primer sets hetRmut-L1(BamHI) & hetRmut-R1 and hetRmut-L2 & hetRmut-R2 (PstI) respectively, fused into a single fragment, digested with BamHI and PstI, and then inserted between the BamHI and PstI sites of pUC18.	This study
pBP1105	A *nrrA* deletion was created using fusion PCR; fragments upstream and downstream of *nrrA* were amplified from pAAWY22574 using primer sets nrrAmut-L1(BamHI) & nrrAmut-R1 and nrrAmut-L2 & nrrAmut-R2 (PstI) respectively, fused into a single fragment, digested with BamHI and PstI, and then inserted between the BamHI and PstI sites of pUC18.	This study
pBP1107	6-kb SacI fragment of pRL2948a cloned into the SacI site of pBP1104.	This study

pBP1108	6-kb SacI fragment of pRL2948a cloned into the SacI site of pBP1105	This study
pBP1193	Inserted 2-kb HindIII fragment from pRL2949a containing the Sp^r cassette into the HindIII site of pMH5, a *modA* integration vector.	This study
pBP1195	Inserted 1.7-kb BglII-SmaI fragment from pBP639 containing the Tc^r cassette between the BglII and SmaI sites of pBP1193.	This study
pBP1197	Inserted 1.1-kb BglII-SacI fragment containing *PnifB2nifB1nifB2* from pSM54 into the 12.2-kb BglII-SacI fragment of pBP1195.	This study
pBP1141	P*coaT:lacZ* fusion was created using fusion PCR; P*coaT* and *lacZ* fragments were amplified from *Synechocystis* sp. PCC 6803 using primer set pcoaR-L(BglII) + pcoaT:lacZ-R and from pBP639 using primer set pcoaT:lacZ-L + lacZ-R(SacI) respectively, fused into a single fragment, digested with BglII and SacI, and then inserted between the BglII and SacI sites of pBP744.	This study
pBP1142	P*coaT:cnfR1* fusion was created using fusion PCR; P*coaT* and *cnfR1* fragments were amplified from *Synechocystis* sp. PCC 6803 using primer set pcoaR-L(BglII) + pcoaT:cnfR1-R and from pBP920 using primer set pcoaT:cnfR1-L + patB1-R(Fus), respectively, fused into a single fragment, digested with BglII and SacI, and then inserted between the BglII and SacI sites of pBP744.	This study
pBP1143	P*coaT:cnfR2* fusion was created using fusion PCR; P*coaT* and *cnfR2* fragments were amplified from *Synechocystis* sp. PCC 6803 using primer set pcoaR-L(BglII) + pcoaT:cnfR2-R and from pBP921 using primer set pcoaT:cnfR2-L + patB2-R(Fus), respectively, fused into a single fragment, digested with BglII and SacI, and then inserted between the BglII and SacI sites of pBP744.	This study
pJU410	*PnifH1:lacZ fusion* vector; Km^r	(3)
pKA6	Source of 2.6-kb *orf-modA* region	(8)
pKM208	Plasmid containing all the genes for recombineering under the P*tac* promoter; *red* and *gam* genes are	(9)

	turned off using *lacI*; and has a temperature-sensitive origin of replication which allows it to be removed from the strain by growing at 37°C.	
pMH2	A 1.4-kb XbaI fragment containing the *modAE* from pKA6 was blunted and inserted into the ScaI site of pJU410 to create a *lacZ* vector that could integrate into the *modAE* region in cyanobacteria.	This study
pMH5	1.1-kb PCR product, containing the Em^r cassette was amplified from pRL2948a using primers Em(BglII)-L + Em(HindIII)-R, digested with BglII and HindIII, and inserted between the BglII and HindIII sites of pMH2.	This study
pRL2948a	Source of mobilization site (*oriT*) and *sacB* gene which confers sucrose sensitivity; Cm^rEm^r	C.P. Wolk
pRL2949a	Source of mobilization site, *oriT*, and *sacB* gene which confers sucrose sensitivity; Sp^r	C.P. Wolk
pRL5801	Source of *aadA* cassette (Sp^rSm^r)	C.P. Wolk
pSM54	Source of hybrid *nifB2:nifB1:nifB2* promoter fused to *lacZ*	(10)
pUC18	Plasmid cloning vector; Ap^r	(11)

Table S3: Oligonucleotides.

Oligonucleotide for cloning	Purpose	Sequence (5'→3') DNA
Em(BglII)-L	Amplifies from the 5' end of the Em^r cassette	ATTAAGATCTCTTCCTTAGCTCCTGAAAATCTCG
Em(HindIII)-R	Amplifies from the 3' end of the Em^r cassette	TATAAAGCTTCGACCTGCATCCCTTAACTTACTTA
FosOriTSp-L2	Amplifies the *oriT* and Sp^r cassette	GGCGTATTTTTTGAGTTATCGAGATTTTCAGGAGCTAAGGAAGCTAAAATAGTGAACGGCAGGTATATGTGATG
FosOriTSp-R2	Amplifies the *oriT* and Sp^r cassette	TTATCACTTATTCAGGCGTAGCAACCAGGCGTTTAAGGGCACCAATAACTCTTGAACGAATTGTTAGACATTATTTGC
hetRmutchk-L	Spans the *hetR* deletion	TACGGAGGAATGAGCATCTCG
hetRmutchk-R	Spans the *hetR* deletion	GTCGCGTTGGCTTAATTCTTG
HetRmut-L1(BamHI)	Amplifies 5' region in *hetR* deletion	ATTAGGATCCCAGCTATTATTAGTGCAAGCGTACC
HetRmut-L2	Amplifies 3' region in *hetR* deletion	TTGTAATATGAGTAACGACATCGATCTGGTGTTTGGTAGAAAAGAAGATTAAGC
HetRmut-R1	Amplifies 5' region in *hetR* deletion	GCTTAATCTTCTTTTCTACCAAACACCAGATCGATGTCGTTACTCATATTACAA
HetRmut-R2(PstI)	Amplifies 3' region in *hetR* deletion	TATACTGCAGCTCATTTACCTTTGGCATTGC
lacZ-R(SacI)	Amplifies the *lacZ* portion of the *PcoaT:lacZ* fusion	CAGTGCAGGAGCTCGTTATCG
nifB2-SmaR2	Extends the *nifB2* region of *pnifB2:nifB1:nifB2*	ATTACCCGGGCAGTTATCTTGTATTTGCTTCAGTTCTTTAG
nrrAmutchk-L	Spans the *nrrA* deletion	TATCAACTGCGCTCTGGACAA
nrrAmutchk-R	Spans the *nrrA* deletion	AAACCAAGCCGATGAAGAATG

nrrAmut-L1(BamHI)	Amplifies 5' region in *nrrA* deletion	TAATGGATCCGGTTTCTATATTCTGAATTTCGACAATC
nrrAmut-L2	Amplifies 3' region in *nrrA* deletion	GTGGGTTCGGTTTGTATTGAAGTGTAGAGGAGGTAGGGAATTAGAG
nrrAmut-R1	Amplifies 5' region in *nrrA* deletion	CTCTAATTCCCTACCTCCTCTACACTTCAATACAAACCGAACCCAC
nrrAmut-R2(PstI)	Amplifies 3' region in *nrrA* deletion	TAATCTGCAGCAAGGAATACAGGTAACATATCCAGT
P1cnfR2fusion1-L	Amplifies *cnfR2*$^{174-1425}$*cnfR1HTH*$^{1420-1590}$ portion of the hybrid fusion in BP950	TGTGATAGCTTGTCCAGTAAAATCCCCAATCCCTTCTCAACCGAAAAAG
P1cnfR2fusion1-R	Amplifies *PcnfR1:cnfR1*$^{1-179}$ portion of the hybrid fusion in BP950	CTTTTTCGGTTGAGAAGGGATTGGGGATTTTACTGGACAAGCTATCACA
P1cnfR2fusion2-L	Amplifies *cnfR2*$^{486-1425}$*cnfR1HTH*$^{1420-1590}$ portion of hybrid fusion in BP951	ATGTAGCAGCCATTGAGGTATTTGATATTAGAGCTGCTTGTATGCACCTGC
P1cnfR2fusion2-R	Amplifies *pcnfR1:cnfR1*$^{1-476}$ portion of hybrid fusion in BP951	GCAGGTGCATACAAGCAGCTCTAATATCAAATACCTCAATGGCTGCTACAT
P1cnfR2fusion3-L	Amplifies *cnfR2*$^{1002-1425}$*cnfR1HTH*$^{1420-1590}$ portion of the hybrid fusion in BP952	GTTATTATTTAAAACCAAAATGGGCAAGGAACAGTGCATTACAATTCCTATGCT
P1cnfR2fusion3-R	Amplifies *PcnfR1:cnfR1*$^{1-992}$ portion of hybrid fusion in BP952	AGCATAGGAATTGTAATGCACTGTTCCTTGCCCATTTTGGTTTTAAATAATAAC
P1patB2-L	Amplifies *cnfR2:cnfR1HTH* portion in the *PcnfR1:cnfR2cnfR1HTH* fusion, BP910	ACGATTAGGGTGGGAAGAAAGTTATATGCCCTACGCCATTACTAATCG

P1patB2-R	Amplifies *PcnfR1* portion in the *PcnfR1:cnfR2cnfR1HTH* fusion, BP910	CGATTAGTAATGGCGTAGGGCATATAACTTTCTTCCCACCCTAATCGT
P2patB1-L	Amplifies *PcnfR2* portion in the *PcnfR2:cnfR1cnfR2HTH* fusion, BP907	CTACTTGAAAATCAGTTGCACAAAACTATGCCTTATACAATTCCTAACAACAGTTG
P2patB1-R	Amplifies *cnfR1:cnfR2HTH* portion in the *PcnfR2:cnfR1cnfR2HTH* fusion, BP907	CAACTGTTGTTAGGAATTGTATAAGGCATAGTTTTGTGCAACTGATTTTCAAGTAG
patB1HTHB2-L	Amplifies *cnfR2HTH* portion in the *PcnfR1:cnfR1:cnfR2HTH* fusion; BP872	CAAAAAATAAAAACCGCAGGTGATCTTTCTACAGAATTTATTATTGAGGCTAGAA
patB1HTHB2-R	Amplifies *PcnfR1:cnfR1HTH*$^{-}$ portion in the *PcnfR1:cnfR1:cnfR2HTH* fusion; BP872	TTCTAGCCTCAATAATAAATTCTGTAGAAAGATCACCTGCGGTTTTTATTTTTTG
patB1L102	Amplifies promoter region in *cnfR1* fusion constructs	TTAGATCTTTTTATCCGGTGAGAAACTTACTA
patB1-R (Fus)	Amplifies the C-terminal end of *cnfR1* fusion constructs	ATTAGAGCTCACGGTTTAACTTATGAAATTGCTGCTTAT
patB2HTHB1-L	Amplifies *cnfR1HTH* portion in the *PcnfR2:cnfR2:cnfR1HTH* fusion; BP873	AGACAAACTTAAAACCGCCAATAACTTTAGTTGGTGAGCAAATTTTGCAAG
patB2HTHB1-R	Amplifies *PcnfR2:cnfR2HTH*$^{-}$ portion in the *PcnfR2:cnfR2:cnfR1HTH* fusion; BP873	CTTGCAAAATTTGCTCACCAACTAAAGTTATTGGCGGTTTTAAGTTTGTCT

patB2-L201	Amplifies promoter region in *cnfR2* fusion constructs	TATTAGATCTGCACTCATCAAGAACCCTATTTAGTTTG
patB2-R(Fus)	Amplifies the C-terminal end of *cnfR2* fusion constructs	ATTAGAGCTCAGGGTGAGGATTTGAATAGTTAATTCTTG
pcoaR-L (BglII)	Amplifies *PcoaT* region in the *PcoaT:*fusions	ATTAAGATCTCTAAAGACAAGTGAGATAGCAGTGGC
pcoaT:cnfR1-L	Amplifies *cnfR1 gene* in the *PcoaT:cnfR1* fusion, BP1142	AGGTAAAAATCCAAGTTAAAAAGCATGCCTTATACAATTCCTAACAACAGTTG
pcoaT:cnfR1-R	Amplifies *PcoaT* region in the *PcoaT:cnfR1* fusion, BP1142	CAACTGTTGTTAGGAATTGTATAAGGCATGCTTTTTAACTTGGATTTTTACCT
pcoaT:cnfR2-L	Amplifies *cnfR2 gene* in the *PcoaT:cnfR2* fusion, BP1143	AGGTAAAAATCCAAGTTAAAAAGCATGCCCTACGCCATTACTAATCG
pcoaT:cnfR2-R	Amplifies *PcoaT* region in the *PcoaT:cnfR2* fusion, BP1143	CGATTAGTAATGGCGTAGGGCATGCTTTTTAACTTGGATTTTTACCT
pcoaT:lacZ-L	Amplifies *lacZ gene* in the *PcoaT:lacZ* fusion, BP1141	AGGTAAAAATCCAAGTTAAAAAGCATGACCATGATTACGGATTCACT G
pcoaT:lacZ-R	Amplifies *PcoaT* region in the *PcoaT:lacZ* fusion, BP1141	CAGTGAATCCGTAATCATGGTCATGCTTTTTAACTTGGATTTTTACCT
pnifB2-BsaL1	Amplifies the *pnifB2:nifB1:nifB2* promoter to extend the *nifB2* region in BP1101	ATGGTCTCATTCTAACATCTGCCACTCCACAA
pnifB2-BsaR1	Amplifies and extends the *nifB2* gene of *pnifB2:nifB1:nifB2* in BP1101	ATGGTCTCAAGAAGTCCTGTAGGTGGTGGTGTC
pnifB2-L1 (JJ)	Amplifies the *pnifB2:nifB1:nifB2* promoter to extend	AGATCTGCTACAAGACGCTTAATCTATTACC

	the *nifB2* region in BP1101	
**Oligonucleotide for qPCR**	**Purpose**	**Sequence (5'→3') DNA**
qcnfR2-mutL	qPCR primer that amplifies the deleted region of *cnfR2*; Fig. 2C	CCCTTGGGAAACTGATGAGTCG
qcnfR2-mutR	qPCR primer that amplifies the deleted region of *cnfR2*; Fig. 2C	TGATGGCTCTGATTGTACGCT
qcnfRg1-R2	qPCR primer that specifically amplifies *pcnfR2:cnfR1* fusion; Fig. S2	GATTTTGATTGCACCCGTAG
qcnfRg2-R1	qPCR primer that specifically amplifies *pcnfR1:cnfR2* fusion; Fig. S2	CCGTCTATAACTTTGATCGCA
qcnfRp1-L1	qPCR primer that specifically amplifies *pcnfR1:cnfR2* fusion; Fig. S2	CATCTCAACGATTAGGGTGG
qcnfRp2-L2	qPCR primer that specifically amplifies *pcnfR2:cnfR1* fusion; Fig. S2	ACGGCTGAATGTTTCCAATA
qhesA2-L	qPCR primer that amplifies the *hesA2* gene; Fig. 6	TTCAATCCGCCGATGTAGCACT
qhesA2-R	qPCR primer that amplifies the *hesA2* gene; Fig. 6	AAGCATCCATCCCATCCATTGC
qnifB1-L	qPCR primer that amplifies the *nifB1* gene; Fig. 1, Fig. 3A&C	TTGCAGACAAAGCCCCAGATA

qnifB1-R	qPCR primer that amplifies the *nifB1* gene; Fig. 1, Fig. 3A&C	CGCCTTCCGCACCTCTATAAC
qnifB2 -L	qPCR primer that amplifies the *nifB2* gene; Fig. 1, Fig. 3B&D, Fig. 5, Fig. 7B	ACACCACCACTATGCACGGATG
qnifB2 -R	qPCR primer that amplifies the *nifB2* gene; Fig. 1, Fig. 3B&D, Fig. 5, Fig. 7B	CCAGGACGGCTTTCATTTGCAC
qnifEN2 -L	qPCR primer that amplifies the *nifEN2* gene; Fig. 6	GTCCCTGATCATTCCACCTCCC
qnifEN2 -R	qPCR primer that amplifies the *nifEN2* gene; Fig. 6	ACACCGAACTACCCATCTGTCG
qnifH2 -L	qPCR primer that amplifies the *nifH2* gene; Fig. 7B	CAAACTGACCATCCCCACCCC
qnifH2 -R	qPCR primer that amplifies the *nifH2* gene; Fig. 7B	ACTACTGCTTTGCCTGCGTCTT
qnifZ2 -L	qPCR primer that amplifies the *nifZ2* gene; Fig. 6	TGACAACATAGCCAACCTCTCCT
qnifZ2 -R	qPCR primer that amplifies the *nifZ2* gene; Fig. 6	ACCAGCATTTGAAATTGGCGCAA
qpatB1-L	qPCR primer that amplifies the deleted region of *cnfR1*; Fig. 2C	GAGAAACGCAAAGACCTCAGC
qpatB1-R	qPCR primer that amplifies the deleted region of *cnfR1*; Fig. 2C	AGCGGCTATTGGTGACTGAAA

qpatB25' -L	qPCR primer that amplifies the 5' end of the *cnfR2* gene; Fig. 7B	TTGCACAAAACTATGCCCTACG
qpatB25' -R	qPCR primer that amplifies the 5' end of the *cnfR2* gene; Fig. 7B	ACACAAACTAGGGTCAATCCAGTAATC

References

1. Gouy M, Guindon S, Gascuel O. 2010. SeaView version 4: A multiplatform graphical user interface for sequence alignment and phylogenetic tree building. Mol Biol Evol 27:221-4.
2. Currier TC, Wolk CP. 1979. Characteristics of *Anabaena variabilis* influencing plaque formation by cyanophage N-1. J Bacteriol 139:88-92.
3. Ungerer JL, Pratte BS, Thiel T. 2010. RNA processing of nitrogenase transcripts in the cyanobacterium *Anabaena variabilis*. J Bacteriol 192:3311-3320.
4. Pratte BS, Thiel T. 2016. Homologous regulators, CnfR1 and CnfR2, activate expression of two distinct nitrogenase gene clusters in the filamentous cyanobacterium *Anabaena variabilis* ATCC 29413. Mol Microbiol 100:1096-109.
5. Thiel T, Lyons EM, Erker JC, Ernst A. 1995. A second nitrogenase in vegetative cells of a heterocyst-forming cyanobacterium. Proc Natl Acad Sci USA 92:9358-9362.
6. Thiel T, Pratte B. 2001. Effect on heterocyst differentiation of nitrogen fixation in vegetative cells of the cyanobacterium *Anabaena variabilis* ATCC 29413. J Bacteriol 183:280-6.
7. Pratte BS, Thiel T. 2014. Regulation of nitrogenase gene expression by transcript stability in the cyanobacterium *Anabaena variabilis*. J Bacteriol 196:3609-3621.
8. Zahalak M, Pratte B, Werth KJ, Thiel T. 2004. Molybdate transport and its effect on nitrogen utilization in the cyanobacterium *Anabaena variabilis* ATCC 29413. Mol Microbiol 51:539-49.
9. Murphy KC, Campellone KG. 2003. Lambda Red-mediated recombinogenic engineering of enterohemorrhagic and enteropathogenic *E. coli*. BMC Molecular Biology 4:11.
10. Vernon SA, Pratte BS, Thiel T. 2017. Role of the nifB1 and nifB2 Promoters in cell-type-specific expression of two Mo nitrogenases in the cyanobacterium *Anabaena variabilis* ATCC 29413. Journal of Bacteriology 199 (4) e00674-16.
11. Vieira J, Messing J. 1982. The pUC plasmids, an M13mp7-derived system for insertion mutagenesis and sequencing with synthetic universal primers. Gene 19:259-268.
